# High-voltage, diffuse delta rhythms coincide with wakeful consciousness and complexity in Angelman syndrome

**DOI:** 10.1093/nc/niaa005

**Published:** 2020-06-14

**Authors:** Joel Frohlich, Lynne M Bird, John Dell’Italia, Micah A Johnson, Joerg F Hipp, Martin M Monti

**Affiliations:** 1 Department of Psychology, University of California Los Angeles, 3423 Franz Hall, Los Angeles, CA, USA; 2 Department of Pediatrics, University of California, San Diego, CA, USA; 3 Division of Genetics/Dysmorphology, Rady Children’s Hospital San Diego, San Diego, CA, USA; 4 Roche Pharma Research and Early Development, Roche Innovation Center Basel, Basel, Switzerland; 5 Department of Neurosurgery, UCLA Brain Injury Research Center, David Geffen School of Medicine, University of California Los Angeles, Los Angeles, CA, USA

**Keywords:** disorders of consciousness, neurology, sleep and dreaming, states of consciousness, theories and models

## Abstract

Abundant evidence from slow wave sleep, anesthesia, coma, and epileptic seizures links high-voltage, slow electroencephalogram (EEG) activity to loss of consciousness. This well-established correlation is challenged by the observation that children with Angelman syndrome (AS), while fully awake and displaying volitional behavior, display a hypersynchronous delta (1–4 Hz) frequency EEG phenotype typical of unconsciousness. Because the trough of the delta oscillation is associated with down-states in which cortical neurons are silenced, the presence of volitional behavior and wakefulness in AS amidst diffuse delta rhythms presents a paradox. Moreover, high-voltage, slow EEG activity is generally assumed to lack complexity, yet many theories view functional brain complexity as necessary for consciousness. Here, we use abnormal cortical dynamics in AS to assess whether EEG complexity may scale with the relative level of consciousness despite a background of hypersynchronous delta activity. As characterized by multiscale metrics, EEGs from 35 children with AS feature significantly greater complexity during wakefulness compared with sleep, even when comparing the most pathological segments of wakeful EEG to the segments of sleep EEG least likely to contain conscious mentation and when factoring out delta power differences across states. These findings (i) warn against reverse inferring an absence of consciousness solely on the basis of high-amplitude EEG delta oscillations, (ii) corroborate rare observations of preserved consciousness under hypersynchronization in other conditions, (iii) identify biomarkers of consciousness that have been validated under conditions of abnormal cortical dynamics, and (iv) lend credence to theories linking consciousness with complexity.

## Introduction

Electroencephalography (EEG) offers a window into neural activity during sleep and wakefulness, generally revealing low-voltage, fast activity during wakefulness and high-voltage, slow activity during non-rapid eye movement (NREM) sleep (Benington *et al.* 1994; Dijk 1995; Cajochen *et al.* 1999; Steriade 2000). The former is typically viewed as informationally rich interactions, whereas the latter is typically viewed as informationally poor synchronization, wherein the number of possible activity states is greatly diminished (Baars *et al.* 2013). Similarly to NREM sleep, loss of consciousness in other states also coincides with a high-voltage EEG rhythm (Alkire *et al.* 2008; Chennu *et al.* 2014; Sitt *et al.* 2014) exhibiting lower signal complexity (Tononi and Edelman 1998; Casali *et al.* 2013; Sarasso *et al.* 2015; Hudetz *et al.* 2016; Schartner *et al.* 2017; Liu *et al.* 2018). For instance, in a state of anesthesia, loss of consciousness coincides with a widespread increase in EEG power at low frequencies (Murphy *et al.* 2011; Supp *et al.* 2011; Lewis *et al.* 2012; Purdon *et al.* 2013), marking a decrease in corticocortical interactions (Mélanie Boly *et al.* 2012; Monti *et al.* 2013). Absence seizures and temporal lobe seizures that impair consciousness are also associated with significant increases in slow waves (Holmes *et al.* 1987; Englot *et al.* 2010). Similar findings have been reported for other modes of loss of consciousness including advanced states of encephalopathy and coma (Kaplan 2004; Sutter and Kaplan 2012), sudden acceleration (Squires *et al.* 1964; Wilson *et al.* 2005), basilar artery migraine (Muellbacher and Mamoli 1994), and convulsive syncope (Varrasi *et al.* 2011).

**Figure 1:**
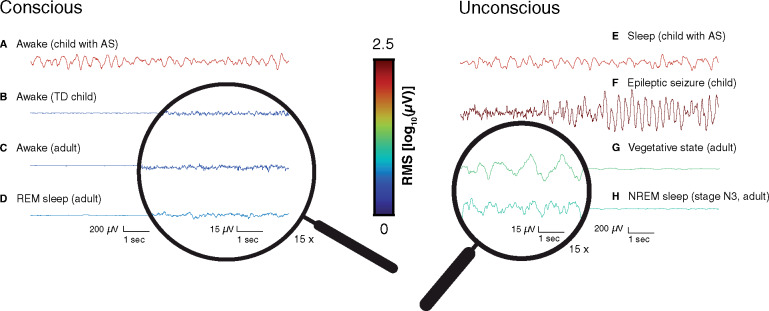
10 s EEG recordings from conscious states (left column) and unconscious states (right column). Voltage traces are color coded by the log_10_ root mean squared, with hotter colors indicating a signal with a higher amplitude and cooler colors indicating a signal with a lower amplitude. A general trend of low-voltage, fast activity is visible in all cases from conscious states except for AS, which paradoxically resembles unconscious EEG activity even during wakeful consciousness (**A**). Panels A and **E** display EEG from a 27-month-old girl with AS included in this study; other panels display data from outside this study with different acquisition, referencing, and preprocessing and are provided for illustrative purposes only. Direct, quantitative comparisons are precluded by these differences. Some EEGs are depicted with amplitude exaggeration (shown inside magnifying glass) to better display waveforms (**B, C, D, G, H**). (A) Awake state EEG (channel Cz) from a 27-month-old girl with AS (Participant 10, Supplementary Table S1) marked by high-amplitude delta oscillations that are more typical of diminished consciousness (cf. right column). This participant did not have seizures and was not taking medication. See additional awake state EEG from this participant in Supplementary Fig. S1B. (B) Awake state EEG (channel Cz) recorded from a typically developing 38-month-old girl. (C) Awake state EEG (bipolar channel F1–F3) recorded from a healthy 37-year-old woman. (D) REM sleep EEG (bipolar channel F1–F3) recorded from a healthy 37-year-old woman. (E) Sleep EEG from a 27-old-girl with AS; note the extreme similarity in waveform to awake state AS EEG in A. See additional asleep state EEG from this participant in Supplementary Fig. S1D. (**F**) Ictal EEG (bipolar channel F3–C3) recorded from a 2-year-old girl with epilepsy during a seizure. (G) Spontaneous EEG (channel Cz) recorded from a 59-year-old man in a vegetative state. (H) Sleep EEG (bipolar channel F1–F3) recorded during NREM (Stage N3) from a healthy 37-year-old woman.

In apparent contradiction to the above data, children with Angelman syndrome (AS) display the rich spectrum of purposeful behavior that implies conscious awareness (as seen here) (Andersen *et al.* 2001; Williams 2005; *What does Angelman Syndrome look like?* 2016) while exhibiting the high-voltage, slow EEG phenotype typical of states of reduced consciousness (Fig. 1, Supplementary Fig. S1; Vendrame *et al.* 2012; Sidorov *et al.* 2017; den Bakker *et al.* 2018; Frohlich *et al.* 2019a; external data in Fig. 1C, D, F, H are from Terzano et al., 2001; Goldberger et al., 2000; Shoeb, 2009). Beyond challenging the general correlation between unconsciousness and cortical hypersynchronization, it may also be argued that the AS EEG phenotype is an enigma from a mechanistic standpoint. While OFF-periods of cortical silence generally occur at the trough of each delta cycle in other contexts (Destexhe *et al.* 2007; Wilson 2008), a different mechanism may occur in AS since it is unclear how consciousness—being dependent on the cerebral cortex (Koch *et al.* 2016)—would persist with cortical neurons offline. Furthermore, if one assumes that neural hypersynchronization is antagonistic to neural complexity (Tononi and Edelman 1998), the AS EEG phenotype may challenge theories of consciousness such as integrated information theory (IIT) that conceptually tie consciousness to functional brain complexity (Oizumi *et al.* 2014; Tononi *et al.* 2016). Specifically, in the case of IIT, the differentiation or diversity of neural activity is a key requirement for consciousness (Oizumi *et al.* 2014). In apparent contradiction to this framework, the awake state AS EEG appears hypersynchronized, with little differentiation visible at the level of the scalp (Vendrame *et al.* 2012; Sidorov *et al.* 2017; den Bakker *et al.* 2018; Frohlich *et al.* 2019a). In translational work, consciousness is also linked to neural complexity by the perturbational complexity index, a successful method of inferring consciousness based on the brain’s electrophysiological “echo” following a magnetic pulse (Casali *et al.* 2013; Comolatti *et al.* 2019). Though an inverse relationship between slow, hypersynchronous EEG activity and complexity is intuitive, it remains uncertain whether this is universally the case.

Although the AS EEG phenotype has long been described in clinical reports (Boyd *et al.* 1988), we are the first to characterize the degree to which the awake EEG in children with AS can support complex dynamics and, moreover, that these dynamics are demonstrably lowered as consciousness decreases during sleep. AS is caused by dysfunction of the gene *UBE3A* (Kishino *et al.* 1997; Buiting *et al.* 2016). Its clinical phenotype encompasses global developmental delay, intellectual disability, microcephaly, epilepsy, and sleep difficulties (Thibert *et al.* 2013; Bird 2014; Buiting *et al.* 2016). Developmental abilities in AS commonly plateau at an age equivalence of less than 30 months (Peters *et al.* 2004; Gentile *et al.* 2010) and the majority of individuals with AS lack functional speech (Williams 1995; Gentile *et al.* 2010); thus, this population presents an unusually clear dissociation between consciousness and other cognitive processes (e.g. language) which often confound efforts to associate a particular quantity with consciousness (Montemayor *et al.* 2019). Puzzlingly, awake state EEG recordings from children with AS display diffuse, slow rhythmic oscillations at delta frequencies (Sidorov et al., 2017; Frohlich *et al.* 2019a) reminiscent of those seen in slow wave sleep and other states of unconsciousness. In fact, spectral power at the delta peak frequency (2.8 Hz) in awake children with AS exceeds that observed in typically developing (TD) children by > 1000% (Frohlich *et al.* 2019a). At face value, either the inverse association between EEG delta power and consciousness does not generalize to the hypersynchronized, but wakeful and conscious, brain in AS, or amidst the pathologically high-voltage slow rhythm observed in awake children with AS, sufficiently complex interactions nonetheless arise and persist over time.

To address this puzzle, we examined whether brain dynamics observed in children with AS during periods of wakefulness were more complex than those observed during periods of sleep despite the global presence of large delta oscillations in both states. As described below, contrary to common readings of EEG and despite diffuse delta oscillations, the awake EEG of children with AS supports significantly greater signal complexity than the asleep AS EEG. This finding persisted even after contrasting periods of wakefulness showing the most pathological EEG signature to the periods of sleep least likely to coincide with any dream experience (Siclari *et al.* 2013, 2017), and, moreover, after accounting for differences in delta power between states.

## Materials and Methods

### Ethics statement

Written consent to participate in the study was obtained from families according to the Declaration of Helsinki and was approved by the institutional review boards of the participating sites.

### Data acquisition

AS is a rare disorder affecting approximately 1 in 10 000–24 000 individuals (Petersen *et al.* 1995; Mertz *et al.* 2013). Given the challenge of obtaining sufficient data from a rare condition, we sought to maximize our sample size through an NIH funded AS Natural History Study [NCT00296764] (i.e. we did not calculate an optimal sample size *a priori*). Spontaneous EEG recordings from children with AS were collected from two sites (Boston Children’s Hospital and Rady Children’s Hospital San Diego) through the natural history study. Participants were encouraged to sleep during part of the EEG acquisition; however, due to severe sleep disturbances in AS (Thibert *et al.* 2013), not all children were able to fall asleep. EEG recordings were acquired in a clinical setting using an international 10 − 20 EEG montage (19 channels). Most participants were on central nervous system medications treating seizures or other symptoms (see Supplementary Table S3 for a complete list of medications and supplements). All EEG data were acquired at one of three native sampling rates: 250 Hz, 256 Hz, or 512 Hz. Annotations denoting sleep, drowsiness, and behavioral state were provided based on behavioral criteria by the EEG technician during data acquisition. Sections of data containing drowsiness were excluded from analysis. Due to the delayed developmental abilities of many children with AS, the EEG acquisition protocol did not control for eye condition (e.g. eyes open or eyes closed) during wakefulness. Some participants gave longitudinal data across multiple visits. Sleep quality data were collected by asking parents how many nights per week their child slept through the night.

From a total of 161 EEG recordings from 99 participants, we identified 35 children with AS whose EEG (48 recordings) contained sections of both sleep and wakefulness. Only participants in the 1–18 years age range were considered, as this is the age range in which the AS delta EEG phenotype has been quantitatively described (Frohlich *et al.* 2019a). Participant details are given in Supplementary Table S1. Only one EEG recording was analyzed per participant according to criteria that included age and amount of usable data. In cases where participants gave data at multiple visits, we analyzed EEG from the visit that yielded the greatest number of valid frequency transform windows at the lowest frequency analyzed (1 Hz) in data sections from the targeted comparison. Ties were broken using the youngest visit, as delta power is known to attenuate with age in AS (Frohlich *et al.* 2019a).

### Preprocessing

Data were imported to MATLAB (The MathWorks, Inc., Torrance, CA, USA) for processing and analysis. We bandpass filtered all recordings 0.5–45 Hz using finite impulse response filtering. Noisy channels and sections of data containing gross artifacts were manually marked to be avoided for purposes of calculating spectral power and signal complexity measures. We also omitted sections of data recorded while participants were exposed to light flash stimuli intended to trigger epileptiform activity. Stereotyped physiological and technical artifacts were removed with independent components analysis using the FastICA algorithm (Hyvarinen 1999; Jung *et al.* 2000). Bad channels were spatially interpolated using a spline interpolation. A prior publication describes the full details of EEG acquisition and preprocessing (Frohlich *et al.* 2019a).

### Wavelet transform

We computed EEG spectral power using a Morlet wavelet transform. We chose a spectral band-width of 1/2 octave (corresponding to *f/σ_f_* ∼ 8.7; *σ_f_*, spectral SD) and spaced the center frequencies logarithmically (base 2) with exponents ranging from 0 (1 Hz) to 5 (32 Hz) (inclusive) in 1/8 octave increments, yielding a total of 41 frequency bins. We then computed power in successive 3/4-overlapping temporal windows of 1 s duration. Time-frequency representations were discarded at time points where the convolution kernel overlapped with sections marked as artifact by more than 20% (see preprocessing). Finally, we averaged the time-frequency representation. To plot spectral power, we first averaged across channels, then log-transformed power before averaging across participants. For statistical comparisons between conditions, we log-transformed power at each point in channel-frequency space (see Statistical Analysis below).

### Signal complexity

Many different quantitative measures of EEG complexity exist, including those that capture the complexity of individual channels (e.g. signal entropy) (Costa *et al.* 2002; Ibáñez-Molina *et al.* 2015) and those that account for interactions between channels (e.g. phi in the context of IIT, or causal density in the context of Granger causality; Seth *et al.* 2011; Toker and Sommer 2019). While the latter category may be more desirable from a theoretical viewpoint, their validity often rests on model assumptions that are difficult to satisfy (e.g. Gaussian or stationary data) (Seth *et al.* 2015; Toker and Sommer 2019). Thus, we opted for the former category and measured EEG signal complexity using two methods, modified multiscale entropy (mMSE) (Costa *et al.* 2002; Xie *et al.* 2008) and generalized Lempel-Ziv (gMLZ) (Lempel and Ziv 1976; Ibáñez-Molina *et al.* 2015; Yeh and Shi 2018) complexity. mMSE compliments gMLZ because multiscale entropy, unlike Lempel-Ziv complexity, does not yield large values for white noise (Costa *et al.* 2002). Specifically, mMSE captures the balance between periodicity and randomness in the signal, computed as modified sample entropy (mSampEn) (Xie *et al.* 2008) across 20 timescales using a coarse graining procedure that excludes high frequencies at each step (Costa *et al.* 2002). gMLZ captures the incompressibility or number of unique substrings in the signal (Lempel and Ziv 1976) and is also computed across 20 timescales (Ibáñez-Molina *et al.* 2015) using two median filters with different smoothing windows to exclude both low and high frequencies at each step (Yeh and Shi 2018). See Table 1 for the center frequencies, smoothing window sizes, and bandwidths at each gMLZ timescale.

**Table 1.** gMLZ timescale parameters

Scale	Center frequency (Hz)	Bandwidth (Hz)	Threshold frequency (Hz)	Smoothing frequency (Hz)	Thresholding window (samples)	Smoothing window (samples)
1	1.00	0.40	0.80	1.20	251	167
2	1.18	0.47	0.95	1.42	211	141
3	1.42	0.58	1.13	1.71	177	117
4	1.68	0.68	1.34	2.02	149	99
5	1.98	0.78	1.57	2.35	127	85
6	2.35	0.95	1.87	2.82	107	71
7	2.82	1.14	2.25	3.39	89	59
8	3.39	1.34	2.74	4.08	73	49
9	3.92	1.48	3.17	4.65	63	43
10	4.65	1.94	3.77	5.71	53	35
11	5.71	2.25	4.65	6.90	43	29
12	6.90	2.59	5.41	8.00	37	25
13	8.00	3.07	6.45	9.52	31	21
14	9.52	4.36	7.41	11.76	27	17
15	11.76	3.81	9.52	13.33	21	15
16	13.33	4.86	10.53	15.38	19	13
17	15.38	6.42	11.76	18.18	17	11
18	18.18	6.84	15.38	22.22	13	9
19	22.22	10.39	18.18	28.57	11	7
20	28.57	17.78	22.22	40.00	9	5

To compute gMLZ (i.e. the difficulty of compressing the signal), we first applied separately two median filters at each timescale: one filter with a smaller kernel (smoothing window) and a second filter with a larger kernel (thresholding window). The output from the first filter is then binarized according to the output from the second filter, which acts as a dynamic threshold. Lempel–Ziv complexity is then computed from the binary timeseries. Smoothing and thresholding windows are both spaced logarithmically to allow for good coverage of the EEG spectrum at all frequency bands, and the difference in size between the smoothing window and the thresholding window is varied to allow larger bandwidth at higher frequencies.

Because the sampling rate influences multiscale analyses, all EEG signals were downsampled to 200 Hz without filtering prior to computing mMSE and gMLZ. Data sections containing artifacts, drowsiness, or light flashes were excised prior to computing mMSE and gMLZ. In the full comparison, mMSE and gMLZ were computed in nonoverlapping segments; in the targeted comparison, we applied 50% overlap between data segments to give better coverage of shorter data. Further details of how mMSE and gMLZ were computed can be found in the Supplementary Methods.

### Comparison of sleep versus wakefulness

Our comparison of data from sleep and wakefulness is informed by the finding that most awakenings from NREM sleep are accompanied by reports of dreams (Stickgold *et al.* 2001) and are thus “contaminated” by consciousness. In addition to the variance in level of consciousness encountered in sleep, there is large variance in delta amplitude encountered during wakefulness in AS children (Sidorov *et al.* 2017). For these two reasons, we performed two comparisons of EEG data: (i) a full comparison using all usable data from both the awake and the asleep state and (ii) a targeted comparison using sections of sleep EEG that are unlikely to coincide with conscious experience (as judged by parietal EEG activity) paired with sections of awake EEG that are especially abnormal as judged by their delta power. For a detailed explanation of how data sections were chosen for the targeted comparison, see Supplementary Methods.

### Statistical analysis

Both mMSE and gMLZ are defined such that regularities (e.g. delta oscillations) in the signal diminish complexity. For this reason, we covaried for delta (1–4 Hz integrated) power using simple linear regression models (separate model for each channel, timescale, and complexity measure). We report results of the targeted comparison both with and without covarying for delta power. To infer whether changes in signal complexity between sleep and wakefulness were mediated by changes in delta power, we used a nonparametric (2 × 10^4^ bootstraps) path analytic framework for mediation analysis (Montoya and Hayes 2017).

To account for a large number of comparisons across channels and timescales or frequencies, we used permutation cluster statistics to test for differences between sleep and wakefulness in both complexity and spectral power (Maris and Oostenveld 2007; Nichols and Holmes 2002). We first performed *t*-tests at each channel and timescale/frequency and then thresholded *t*-statistics using *P* = 0.01 before clustering in channel-timescale space (complexity) or channel-frequency space (spectral power). For each cluster, we then derived two-tailed statistical significance nonparametrically by permuting the condition labels 10^4^ times and comparing the size of the original cluster to the empirical distribution of cluster sizes. This approach is unbiased with respect to directionality, frequency/timescale, and electrode location. In total, we performed eight separate tests: power and complexity were examined in both a full comparison and a targeted comparison (three EEG measures × two comparisons), and complexity was also examined in a follow-up targeted comparison in which we covaried for delta power (two EEG measures × one comparison). We then adjusted for the total number of tests using a Bonferroni correction, yielding α = 0.0063. Effect sizes for each cluster (median across all cluster members) were measured as Cohen’s *d*.

## Results

Herein, we have assessed the degree to which signal complexity can emerge from the pathologically hypersynchronous brain dynamics typical of children with AS and, specifically, whether such dynamics differ significantly with relative level of consciousness (i.e. wake, sleep). We first addressed this question by analyzing all usable EEG data (henceforth, full comparison). We then repeated the analysis while controlling for two possible confounders (henceforth, targeted comparison). All comparisons were within-subject and contrasted awake and asleep EEG recorded from the same EEG session.

Our sample included a cohort of 35 children with AS (15 female), ranging from 13 to 130 months of age (mean ± SD = 47.9 ± 28.6 months), of which 25 had a deletion of chromosome 15q11-q13 and the remaining 10 had other genetic aberrations affecting *UBE3A* (see Supplementary Table S1 for individual demographic details and length of EEG data used in each of the analyses). Qualitative inspection of EEG recordings revealed strongly abnormal EEG patterns in both sleep and wakefulness (see Fig. 1A and E and Supplementary Fig. S1 for examples from a participant without seizures or medications).

### Full comparison

#### Full comparison: EEG delta and alpha/beta power are modulated by sleep in AS

**Figure 2:**
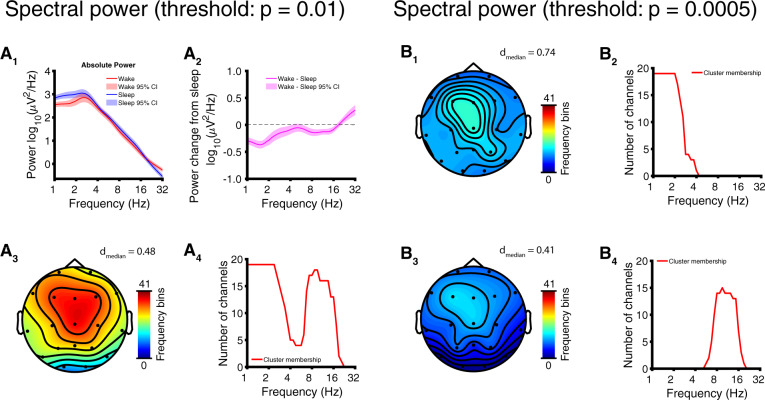
Full comparison: EEG delta and alpha/beta power are modulated by sleep in AS. Results depicted here reflect within-subject comparisons of sleep versus wakefulness. (**A**) EEG spectral power with t-statistics thresholded at *P* = 0.01 for permutation cluster statistics. (**A_1_**) Channel and participant averaged power spectrum (log-scaled, mean ± 95% CI) and (**A_2_**) wake − sleep change (mean ± 95% CI). (**A_3_**) Channel-space profile of cluster (*P* < 10^−4^) of decreased power in wakefulness (color coded by the number of frequency bins participating in the cluster at each channel). (**A_4_**) Frequency-space profile of cluster (i.e. number of channels participating at each frequency bin; note that this cluster fuses high-frequency and low-frequency aspects). (**B**) Cluster in (A) broken into separate low-frequency and high-frequency subclusters by applying a stricter threshold (*P* = 0.0005) to t-statistics. (**B_1_**) Channel-space profile of delta-frequency subcluster (decreased power in wakefulness). (**B_2_**) Frequency-profile of subcluster showing participation at delta frequencies. (**B_3_**) Channel-space profile of alpha-/beta-frequency subcluster (increased power in wakefulness). (**B_4_**) Frequency-profile of subcluster showing participation at alpha/beta frequencies.

The full comparison revealed peaks in the delta band for both the awake and asleep condition (channel averaged), with a sharper peak in the awake state and a broader peak in the asleep state (Fig. 2A; see Supplementary Fig. S1A for visualization of the untransformed power). The duration of usable EEG ranged from 3.39 to 167 min (awake state, mean ± SD = 16.7 ± 27.1 min) and 2.89 to 123 min (asleep state, mean ± SD = 17.3 ± 20.1 min); high variance in duration of usable data is likely attributable to the variability of developmental abilities and behavioral phenotype in participants. Differences in EEG measures between the asleep and awake state were revealed by clusters of channels and frequency bins (power) or timescales (complexity) showing similar modulation. Power was generally decreased in wakefulness at frequencies under 20 Hz, with the largest decrease occurring as a 53.0% reduction at *f* = 1.52 Hz (Supplementary Fig. S2B). These changes in wakeful power mapped onto a significant cluster (*P* < 10^−4^, cluster permutation test) in channel-frequency space with a spatially diffuse topography that was largest over frontocentral areas (Fig. 2A3,4; effect size: *d* = −0.48 ± 0.26, median ± SD; see Table 2 for full details of clusters). Because the spectral profile of this cluster appeared to “fuse” an oscillatory change in the delta frequency range with an oscillatory change in the alpha-/beta-frequency range (Fig. 2A4), we then repeated the cluster randomization statistics with a stricter threshold (*P* = 0.0005) to observe the topography of each oscillatory change separately (Fig. 2B, Supplementary Table S2). We also observed a >100% increase in power at high frequencies (*f* > 28 Hz) in the awake state relative to the asleep state (Fig. 2A2, Supplementary Fig. S2B); however, the corresponding cluster did not reach statistical significance (Cluster 4, Table 2). See Supplementary Fig. S3 for topographic plots of power by frequency band.

**Figure 3:**
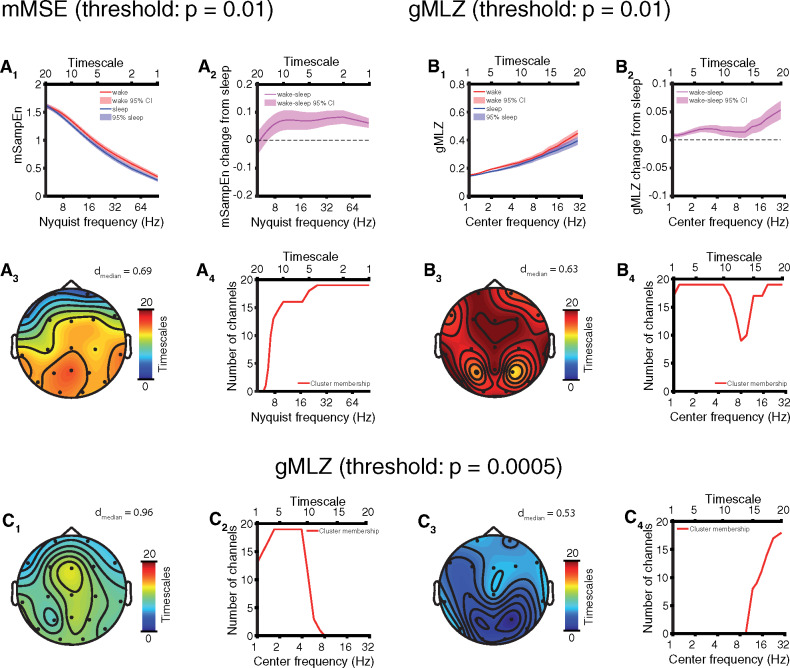
Full comparison: EEG complexity is modulated by sleep in AS. Results depicted here reflect within-subject comparisons of sleep versus wakefulness. (**A**) mMSE with t-statistics thresholded at *P* = 0.01 for permutation cluster statistics. (**A_1_**) Channel and participant averaged (mean ± 95% CI) SampeEn and (**A_2_**) wake − sleep change (mean ± 95% CI). (**A_3_**) Channel-space profile of cluster (*P* = 0.0007) of increased mMSE in wakefulness. (**A_4_**) Timescale-space profile of cluster showing greater participation at fast timescales. (**B**) gMLZ with t-statistics thresholded at *P* = 0.01 for permutation cluster statistics. (**B_1_**) Channel and participant averaged (mean ± 95% CI) gMLZ and (**B_2_**) wake − sleep change (mean ± 95% CI). (**B_3_**) Channel-space profile of cluster (*P* < 10^−4^) of increased gMLZ in wakefulness. (**B_4_**) Timescale-space profile of cluster fusing low-frequency and high-frequency aspects. (**C**) Cluster in (B) broken into separate low-frequency and high-frequency subclusters by applying a stricter threshold (*P* = 0.0005) to t-statistics. (**C_1_**) Channel-space profile of low-frequency subcluster. (**C_2_**) Timescale-space profile of low-frequency subcluster. (**C_3_**) Channel-space profile of high-frequency subcluster. (**C_4_**) Timescale-space profile of high-frequency subcluster.

**Figure 4:**
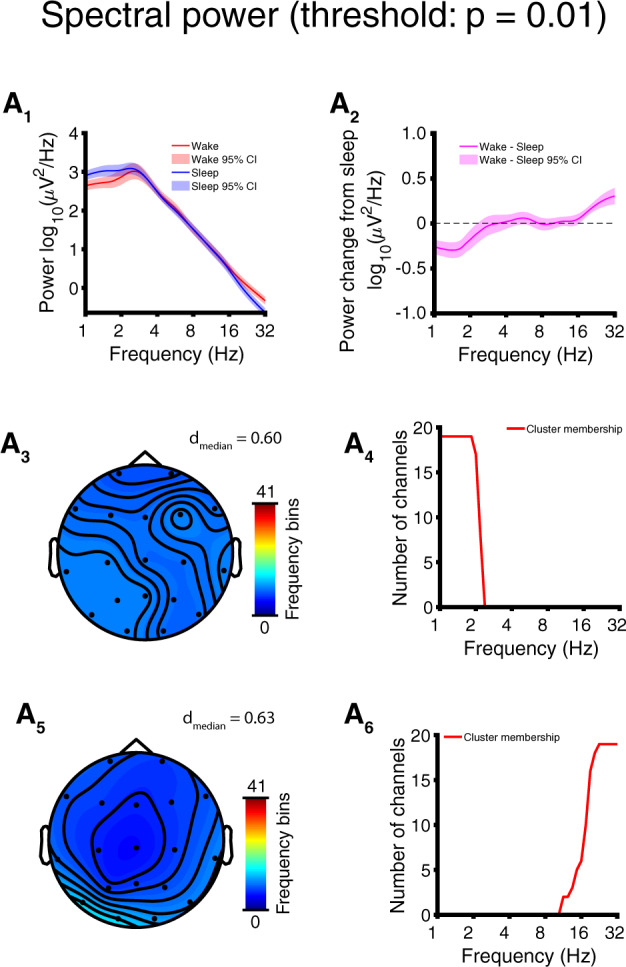
Targeted comparison: EEG low delta power is modulated by sleep in AS. Results depicted here reflect within-subject comparisons of sleep versus wakefulness. (**A**) EEG spectral power with t-statistics thresholded at *P* = 0.01 for permutation cluster statistics. (**A_1_**) Channel and participant averaged power spectrum (log-scaled, mean ± 95% CI) and (**A_2_**) wake − sleep change (mean ± 95% CI). (**A_3_**) Channel-space profile of low delta-frequency cluster (*P* = 0.0011) of decreased power in wakefulness (color coded by the number of frequency bins participating in the cluster at each channel). (**A_4_**) Frequency-space profile of low delta-frequency cluster (i.e. number of channels participating at each frequency bin). (**A_5_**) Channel-space profile of beta-frequency cluster (*P* = 0.0022) of increased power in wakefulness revealing a topography suggestive of muscle artifact. (**A_6_**) Frequency-space profile of beta-frequency cluster; participation at high frequencies is suggestive of muscle artifact.

**Table 2:** Channel-frequency (power) and channel-timescale (complexity) clusters identified using permutation cluster statistics

	EEG measure	Comparison	Direction (awake- sleep)	Regressed delta power?	*P*-value	Cohen’s *d* (cluster median)	Cohen’s *d* (cluster SD)	Cluster size	Percentage	Low freq	High freq	Minimum number of channels	Maximum number of channels
**Cluster 1**	**gMLZ**	**Full**	**Increase**	**No**	**<10^−4^**	**0.63**	**0.32**	**345**	**90.79**	**1.0**	**28.6**	**9**	**19**
**Cluster 2**	**Power**	**Full**	**Decrease**	**No**	**<10^−4^**	**−0.48**	**0.26**	**486**	**62.39**	**1.0**	**20.7**	**1**	**19**
**Cluster 3**	**mMSE**	**Full**	**Increase**	**No**	**0.0007**	**0.69**	**0.12**	**231**	**60.79**	**6.3**	**100.0**	**1**	**19**
Cluster 4	Power	Full	Increase	No	0.017	0.56	0.26	83	10.65	19.0	32.0	3	19
**Cluster 5**	**gMLZ**	**Targeted**	**Increase**	**No**	**<10^−4^**	**0.64**	**0.32**	**279**	**73.42**	**1.0**	**28.6**	**1**	**19**
**Cluster 6**	**mMSE**	**Targeted**	**Increase**	**No**	**0.001**	**0.67**	**0.11**	**226**	**59.47**	**6.3**	**100.0**	**3**	**19**
**Cluster 7**	**Power**	**Targeted**	**Decrease**	**No**	**0.0011**	**−0.60**	**0.19**	**177**	**22.72**	**1.0**	**2.2**	**8**	**19**
**Cluster 8**	**Power**	**Targeted**	**Increase**	**No**	**0.0022**	**0.63**	**0.24**	**157**	**20.15**	**11.3**	**32.0**	**2**	**19**
Cluster 9	Power	Targeted	Increase	No	0.1727	0.24	0.02	4	0.51	4.8	6.2	1	1
Cluster 10	Power	Targeted	Increase	No	0.2021	0.27	0.02	3	0.39	4.8	5.7	1	1
Cluster 11	Power	Targeted	Increase	No	0.2448	0.25	0.05	2	0.26	5.2	5.2	2	2
Cluster 12	Power	Targeted	Increase	No	0.3036	0.24	N/A	1	0.13	4.0	4.0	1	1
**Cluster 13**	**gMLZ**	**Targeted**	**Increase**	**Yes**	**<10^−4^**	**2.42**	**1.28**	**361**	**95.00**	**1.0**	**28.6**	**10**	**19**
**Cluster 14**	**mMSE**	**Targeted**	**Increase**	**Yes**	**<10^−4^**	**1.92**	**0.81**	**346**	**91.05**	**5.0**	**100.0**	**11**	**19**
Cluster 15	gMLZ	Targeted	Decrease	Yes	0.0844	**−**0.92	N/A	1	0.26	1.0	1.0	1	1
Cluster 16	gMLZ	Targeted	Decrease	Yes	0.0844	**−**0.58	N/A	1	0.26	1.0	1.0	1	1
Cluster 17	gMLZ	Targeted	Decrease	Yes	0.0844	**−**0.71	N/A	1	0.26	1.0	1.0	1	1

Bold rows are clusters reported in the text and figures that meet statistical significance after a Bonferroni correction (α = 0.0063). *P*-values are derived from empirical cluster size distributions using cluster permutation tests. Effect sizes are reported as Cohen’s *d* (median and standard deviation across all cluster points; standard deviation is reported as N/A for clusters with only 1 point).

### Full comparison: EEG complexity is modulated by sleep in AS

We next examined mMSE and gMLZ and found greater EEG signal complexity in the awake state as compared to sleep. Specifically, the channel averaged mSampEn decreased monotonically with faster timescales, but was greater during wakefulness as compared to sleep (Fig. 3A1,2), with the exception of frequencies ≤ 6.25 Hz (i.e. the five slowest timescales). Greater mMSE during wakefulness was marked by a significant cluster (*P* = 0.0007, cluster permutation test) covering all channels but largest over central and posterior areas (Fig. 3A3,4; effect size: *d* = 0.69 ± 0.12, median ± SD). By comparison, gMLZ increased monotonically with faster timescales and was larger in wakefulness as compared to sleep at all timescales, particularly those with center frequencies corresponding to delta and beta frequencies (channel-averaged; Fig. 3B1,2). These changes were accompanied by a significant cluster (*P* < 10^−4^, cluster permutation test) encompassing 90.8% of channel-timescale space (Fig. 3B3,4; effect size: *d* = 0.63 ± 0.32, median ± SD). The cluster appeared to fuse complexity changes corresponding to fast and slow timescales. We thus repeated the analysis, applying a stricter threshold (*P* = 0.0005) to t-statistics to view the topography of each change separately (Fig. 3C). Effect sizes in the low-frequency subcluster were large (*d* = 0.96 ± 0.27; Fig. 3C1, Supplementary Table S2). See Supplementary Fig. S4 for complete visualizations of all clusters and Supplementary Fig. S5 for visualizations of individual participants’ data. Participant genotype (deletion or non-deletion) significantly correlated with mMSE and gMLZ at some timescales (Supplementary Fig. S6). This covariate was fully controlled for by the within-subject design of our study.

### Targeted comparison

To account for both the large variance in delta amplitude in awake children with AS and the possibility of conscious mentation during NREM sleep, we reinforced our full comparison with a targeted comparison that accounted for the above confounders (i.e. delta variability and conscious mentation) as follows. We addressed the fromer concern by focusing only on the segments of the most abnormal awake state EEG, as operationalized by delta power. We then addressed the latter concern by contrasting the pathological awake state EEG with the periods of asleep state EEG that are the least likely to correspond to any dream experience, as operationalized by the ratio of parietal delta power to high-frequency power (Siclari *et al.* 2013, 2017), where higher values of this ratio correspond to a greater probability of unconsciousness. In other words, we compared the most abnormal segments of EEG still corresponding, nonetheless, to a state of wakeful consciousness, to the segments of sleep EEG least likely to correspond to any conscious experience. By using only these segments, one may expect the findings from the full comparison to disappear if EEG complexity is not always greater during wakefulness as compared to dreamless sleep, e.g., during bouts of especially high-amplitude delta in wakefulness (Sidorov *et al.* 2017). The duration of selected EEG ranged from 2.07 to 9.44 min (awake state, mean ± SD = 3.96 ± 1.67 min) and 1.48 to 5.32 min (asleep state, mean ± SD = 3.24 ± 1.02 min).

### Targeted comparison: EEG low delta power is modulated by sleep in AS

The targeted awake EEG sections were characterized by a prominent peak in the delta band, while the targeted asleep EEG sections were characterized by two delta band peaks at different octaves (channel averaged; Fig. 4A1), which are best visualized in the untransformed power (Supplementary Fig. S7A); this suggests the presence of two separate oscillatory processes, one related to sleep and one related more specifically to AS pathology. Decreases in power between the two states were restricted to the delta band (max change: 41.3% decrease at *f* = 1.34 Hz), with >100% increases also occurring at high frequencies (*f* > 25 Hz) (Fig. 4A2, Supplementary Fig. S7B). Permutation cluster statistics identified two small but significant clusters differing between the two states. The first cluster corresponded to decreased power at low delta (1.0–2.2 Hz) frequencies in the awake state (*P* = 0.0011, cluster permutation test) and lacked a distinct scalp topography (Fig. 4A3,4; effect size: *d* = −0.60 ± 0.19, median ± SD). The second cluster corresponded mostly to increased power at largely beta (11.3–32 Hz) frequencies in the awake state (*P* = 0.0022, cluster permutation test) and displayed a scalp topography suggestive of neck muscle artifact (Fig. 4A5,6; effect size: *d* = 0.63 ± 0.24, median ± SD). These results show that, after accounting for the confounders that motivated our targeted comparison, the most reliable spectral differences between sleep and wakefulness were found at low delta frequencies. See Supplementary Fig. S8 for topographic plots of power by frequency band.

**Figure 5:**
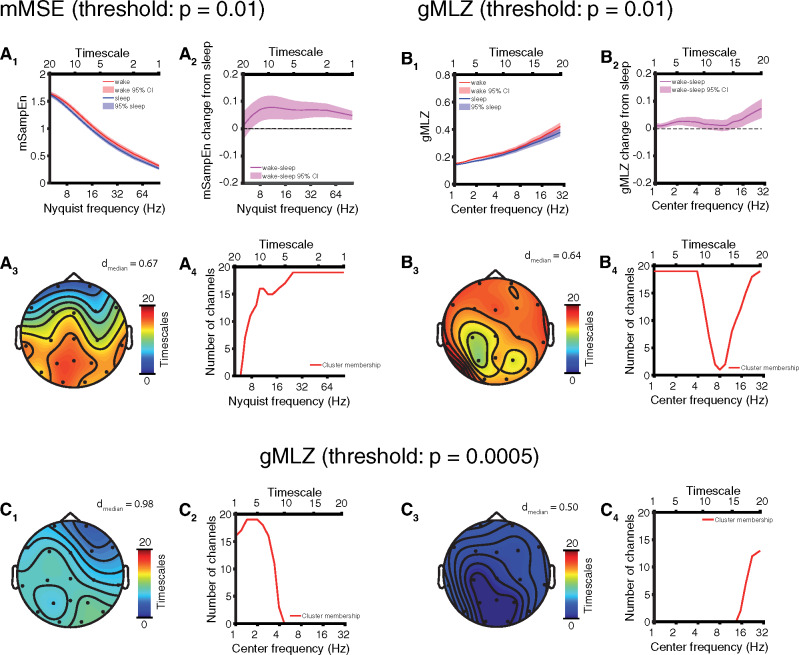
Targeted comparison: EEG complexity is modulated by sleep in AS. Results depicted here reflect within-subject comparisons of sleep versus wakefulness. (**A**) mMSE with t-statistics thresholded at *P* = 0.01 for permutation cluster statistics. (**A_1_**) Channel and participant averaged (mean ± 95% CI) SampeEn and (**A_2_**) wake − sleep change (mean ± 95% CI). (**A_3_**) Channel-space profile of cluster (*P* = 0.001) of increased mMSE in wakefulness. (**A_4_**) Timescale-space profile of cluster showing greater participation at fast timescales. (**B**) gMLZ with t-statistics thresholded at *P* = 0.01 for permutation cluster statistics. (**B_1_**) Channel and participant averaged (mean ± 95% CI) gMLZ and (**B_2_**) wake − sleep change (mean ± 95% CI). (**B_3_**) Channel-space profile of cluster (*P* < 10^−4^) of increased gMLZ in wakefulness. (**B_4_**) Timescale-space profile of cluster fusing low-frequency and high-frequency aspects. (**C**) Cluster in (B) broken into separate low-frequency and high-frequency subclusters by applying a stricter threshold (*P* = 0.0005) to t-statistics. (**C_1_**) Channel-space profile of low-frequency subcluster. (**C_2_**) Timescale-space profile of low-frequency subcluster. (**C_3_**) Channel-space profile of high-frequency subcluster. (**C_4_**) Timescale-space profile of high-frequency subcluster.

**Figure 6:**
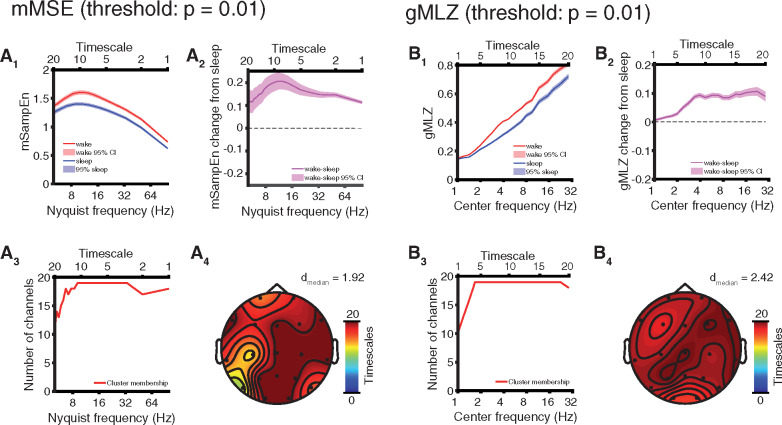
Targeted comparison: EEG complexity changes are robust to covarying for delta power. (**A**) mMSE with t-statistics thresholded at *P* = 0.01 for permutation cluster statistics. (**A_1_**) Channel and participant averaged (mean ± 95% CI) SampeEn and (**A_2_**) wake − sleep change (mean ± 95% CI). (**A_3_**) Channel-space profile of cluster (*P* < 10^−4^) of increased mMSE in wakefulness (color coded by the number of frequency bins participating in the cluster at each channel). (**A_4_**) Timescale-space profile of cluster (i.e. number of channels participating at each frequency bin; this cluster showed high participation across all timescales). (**B**) gMLZ with t-statistics thresholded at *P* = 0.01 for permutation cluster statistics. (**B_1_**) Channel and participant averaged (mean ± 95% CI) gMLZC and (**B_2_**) wake − sleep change (mean ± 95% CI). (**B_3_**) Channel-space profile of cluster (*P* < 10^−4^) of increased gMLZ in wakefulness. (**B_4_**) Timescale-space profile of cluster showing high participation across all timescales.

### Targeted comparison: EEG complexity is modulated by sleep in AS

Analysis of EEG signal complexity in the targeted comparison revealed similar results to the full comparison (channel-averaged; Fig. 5A1,2). Greater mMSE in wakefulness was marked by a significant cluster (*P* = 0.001, cluster permutation test) exhibiting similar topography to the corresponding cluster found in the full comparison (Fig. 5A3,4; effect size: *d* = 0.67 ± 0.11, median ± SD). The gMLZ curves also exhibited the same behavior seen in the full comparison (channel-averaged; Fig. 5B1,2) and yielded a significant cluster (*P* < 10^−4^, cluster permutation test) marking greater complexity during wakefulness (Fig. 5B3,4; effect size: *d* = 0.64 ± 0.32, median ± SD). The cluster appeared to fuse complexity changes corresponding to fast and slow timescales. We thus repeated the analysis, applying a stricter threshold (*P* = 0.0005) to t-statistics to view the topography of each change separately. Effect sizes in the low-frequency subcluster were large (*d* = 0.98 ± 0.20, median ± SD; Fig. 5C, Supplementary Table S2). See Supplementary Fig. S9 for complete visualizations of all clusters and Supplementary Fig. S10 for visualizations of individual participants’ data. Participant genotype and log_2_(age) significantly correlated with mMSE and gMLZ at some timescales (Supplementary Fig. S11). These covariates were fully controlled for by the within-subject design of our study.

### Effects of sleep on EEG complexity are not mediated by delta power

As shown in Fig. 4A1, despite having selected the most pathological segments of the awake EEG dataset, delta power still differed significantly across wakefulness and sleep. Delta power was also negatively correlated with mSampEn at most timescales after averaging across channels, explaining the majority of the variance in mSampEn at fast timescales (*R*^2^ > 0.5 for Nyquist frequency ≥ 20 Hz, awake, and Nyquist frequency ≥ 50 Hz, asleep, Supplementary Fig. S12A). To account for the possibility that greater regularity (and thus lower complexity) is introduced in sleep merely by greater delta power, we performed a mediation analysis to infer whether changes in complexity were mediated by changes in delta power. Delta power did not mediate the effect of state (i.e. wakefulness versus sleep) on mMSE (Supplementary Fig. S12B, *P* > 0.05 all timescales, uncorrected). Even more so than mMSE, delta power was negatively correlated with gMLZ at most timescales (*R*^2^ > 0.5 for center frequency ≥ 2.82 Hz, asleep and awake state, Supplementary Fig. S12C). Yet again, delta power did not mediate the effect of state on gMLZ (Supplementary Fig. S12D, *P* > 0.05 all timescales, uncorrected). Given the observed negative relationship between delta power and complexity measures, in what follows we repeated the targeted comparison after covarying for delta power (integrated 1 – 4 Hz).

### Targeted comparison: EEG complexity changes are robust to covarying for delta power

Finally, as shown in Fig. 6, our overall findings remained unchanged after controlling for delta power differences across wakefulness and sleep by regressing out 1 − 4 Hz power. Specifically, while mMSE curves were no longer monotonic with timescale, they still show the awake EEG to be more complex than the asleep EEG. The largest increase from sleep occurred at low frequencies (i.e. the fastest timescale; channel-averaged; see Fig. 6A1,2). This relative increase in mMSE during wakefulness corresponded to a significant (*P* < 10^−4^, cluster permutation test) and nearly saturated cluster (Fig. 6A3,4; effect size: *d* = 1.92 ± 0.81, median ± SD). With respect to gMLZ, covarying for delta power again leads to the same qualitative result reported above, with wakefulness showing consistently greater complexity than sleep. Intriguingly, however, the divergence in complexity between the two states is even greater after factoring out delta power, with the largest increase from sleep occurring at the center frequency of 22.2 Hz (i.e. the 19th timescale; channel averaged; see Fig. 6B1,2). The relative increase in gMLZ during wakefulness corresponds to a significant (*P* < 10^−4^, cluster permutation test) and, again, nearly saturated cluster (Fig. 6B3,4; effect size: *d* = 2.42 ± 1.28, median ± SD). See Supplementary Fig. S13 for complete visualizations of all clusters and Supplementary Fig. S14 for visualizations of individual participants’ data.

## Discussion

Children with AS exhibit an EEG phenotype resembling states of diminished consciousness in typical individuals, while also exhibiting purposeful behavior consistent with a state of wakeful awareness, albeit marked by severe intellectual disability. This paradoxical EEG pattern during conscious wakefulness, together with similar circumstances occasionally observed in non-convulsive status epilepticus (Gökyiǧit and Çalişkan 1995), Rett syndrome (Laan and Vein 2002), postoperative delirium (Palanca *et al.* 2017), schizophrenia (Matsuura *et al.* 1994), and immediately following tracheal intubation for general anesthesia (Gaskell *et al.* 2017), challenges the view that high-voltage, slow EEG activity is a reliable marker of loss of consciousness.

At face value, the pathological presence of slow, high-amplitude oscillations during a state of wakeful awareness appears problematic for theoretical frameworks linking consciousness to complexity (Tononi and Edelman 1998; Oizumi *et al.* 2014; Tononi *et al.* 2016). This is because the hypersynchronization needed to produce such slow, high-voltage rhythms limits the degrees of freedom on neuronal populations; neurons are entrained to their neighbors, reducing the diversity of cortical activity. With a smaller repertoire of cortical states, the brain exhibits less functional differentiation (Koch *et al.* 2016) and thus lacks one of the two cardinal elements theoretically needed for a system to be conscious: differentiation and integration (Tononi and Edelman 1998; Oizumi *et al.* 2014; Tononi *et al.* 2016). Yet, the data presented above suggest that even in the presence of pathological, global slowing and synchronization of brain dynamics, the complexity of scalp EEG signals still emerges and systematically varies with levels of consciousness. It thus remains an open question whether high-voltage delta rhythms are inversely related to complexity and consciousness in the manner that has been previously assumed (Tononi and Edelman 1998), with at least one recent review on the topic acknowledging that slow waves may occur during consciousness (Koch *et al.* 2016). The relationship in question is also cast into doubt by recent EEG work with the psychedelic *N,N*-dimethyltryptamine (DMT) in healthy adult participants showing that both elevated Lempel–Ziv complexity and phenomenologically rich, visual experiences coincide with low-frequency EEG rhythmicity induced by DMT (Timmermann *et al.* 2019).

Our interpretation of our findings withstands two possible criticisms. First, one may argue that the abnormal EEG phenotype in AS is explained entirely by intellectual disability. From this perspective, the EEG phenotype of awake, behaving children with AS is trivial. However, this perspective would fail to explain how purposeful behaviors that demonstrate consciousness to an observer arise in AS amidst global, high-amplitude delta oscillations that should be expected to diminish consciousness through cortical down-states. (Franks 2008; Rosanova *et al.* 2018). The fact that children with AS have intellectual disability does nothing to resolve the foregoing paradox given their volitional behavior under these unusual circumstances. In fact, the presence of intellectual disability in AS may strengthen our findings, because differences between sleep and wakefulness are less likely to track cognitive processes such as language—which are severely impaired in AS (Williams 1995; Gentile *et al.* 2010)—that may otherwise confound studies of consciousness (Montemayor *et al.* 2019). Incidentally, while cortical hyperexcitability may indeed relate to intellectual disability in AS, similar levels of intellectual impairment occur without high-amplitude delta activity in duplication 15q11.2-q13.1 syndrome, in which the *UBE3A* locus is duplicated rather than deleted (Frohlich *et al.* 2016, 2019b).

Other criticism of our interpretation may come from the perspective that our findings are trivial in so far as EEG activity reflects not only conscious brain activity but also subconscious sensorimotor processing. From this perspective, one may argue EEG complexity should always scale with the degree of sensory input and motor output regardless of conscious state. However, when conscious processes go offline and subconscious sensorimotor processing—including auditory and somatosensory processing (Portas *et al.* 2000; Massimini *et al.* 2003), semantic processing, (Brualla *et al.* 1998; Ibáñez *et al.* 2006), implicit vocabulary learning (Züst *et al.* 2019), and ambulatory behavior (Zadra *et al.* 2004)—is preserved during NREM sleep, EEG activity switches from complex, low-voltage, fast activity characterized by high effective connectivity during wakefulness to uniform, high-voltage, slow activity characterized by low effective connectivity during NREM sleep (Tononi and Edelman 1998; Massimini *et al.* 2005). This switch demonstrates that subconscious sensorimotor processing may occur in the absence of electrophysiological complexity.

### EEG biomarkers of consciousness in AS

For each of three candidate biomarkers of consciousness tested (spectral power, mMSE, and gMLZ), we found significant clusters that differentiate sleep from wakefulness in AS. Significant clusters were found regardless of whether we performed a full comparison of all usable data or a targeted comparison of those EEG sections that were least likely to coincide with dream experiences (sleep state) or were especially abnormal as judged by delta EEG power (awake state). The largest within cluster/subcluster effect sizes we observed (without shrinking within condition variance by regressing out delta power) were those belonging to a low-frequency complexity change in the gMLZ cluster [*d*_median_ = 0.96/0.98 (full comparison/targeted comparison), Figs 3C1,2 and 5C1,2, Supplementary Table S2]. These effect sizes surpassed those observed for spectral power, even when considering low-frequency changes encompassed by the power cluster/subcluster with the largest effects [*d*_median_ = 0.74/0.60 (full comparison/targeted comparison), Figs 2B1,2 and 4A3,4, Table 2, Supplementary Table S2]. Topographic visualizations of mMSE clusters (Figs 3A3 and 5A3) revealed greater involvement in posterior regions, suggesting the importance of a posterior hot zone in wakeful consciousness (Melanie Boly *et al.* 2017). On the other hand, gMLZ clusters showed nearly uniform involvement across the entire scalp (Figs 3B3 and 5B3), suggesting that gMLZ may have greater sensitivity than mMSE to frontal changes in complexity.

Although our study found that delta power was indeed modulated by diminished consciousness (i.e. sleep) in AS, the effect sizes in both comparisons (*d*_median_ = 0.60 – 0.74) were a fraction of that yielded by a prior comparison of delta power between TD control children and children with AS in the awake state (*d* = 1.22) (Frohlich *et al.* 2019a). Expressed as a percent change referenced to wakefulness, delta power increases with sleep in AS by a maximum of 162% at *f* = 1.5 Hz (mean across channels and participants, full comparison), whereas delta power in AS is greater in the awake state relative to TD control children by 1182% (Frohlich *et al.* 2019a). Thus, the difference between groups during wakefulness is an order of magnitude greater than the difference within AS with sleep/wakefulness. Because the variance within AS between conscious states is much smaller than the variance between AS and TD control children, caution should be applied when using delta EEG power alone as a biomarker for consciousness.

### Neural hypersynchronization in AS

**Figure 7:**
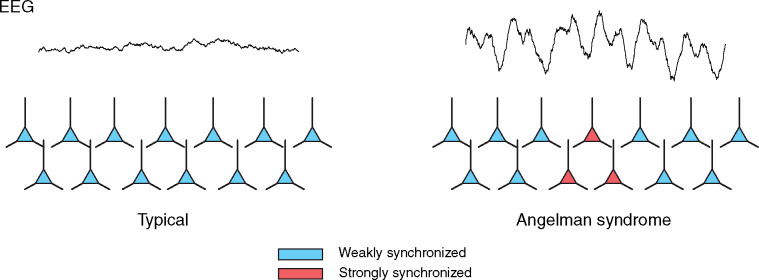
Hypothetical model of cortical hypersynchronization in AS. A small number of hypersynchronized cortical oscillators may explain the AS EEG phenotype. In the typically developing brain (left), synchronization between cortical pyramidal cells is relatively weak (blue; each cartoon pyramidal cell represents a neuronal population composed of millions of actual pyramidal cells). As a result, neuronal populations are able to oscillate relatively independently; this allows for a large repertoire of cortical states and a corresponding complex, low amplitude EEG. By comparison, in AS (right), a small number of neuronal populations (red) are likely hypersychronized. Although these hypersychronized neurons are a minority, their activity is more easily detected from the scalp due to their greater coordination. As a result, the AS EEG is dominated by high amplitude, slow activity from local generators that project globally to the scalp by volume conduction. This is analogous to a small group of people in a sports area whose synchronized chanting drowns out the uncoordinated chatter of the larger majority. Because the weakly synchronized neuronal populations in AS are still free to oscillate relatively independent of each other, the AS brain still enjoys the large repertoire of brain states theoretically necessary for consciousness.

Our results clearly indicate that despite the appearance of global hypersynchronization in AS, there remains sufficient information-rich activity to allow the complex dynamics typical of conscious awareness to arise. Given this observation, how do complex brain dynamics and consciousness emerge against a background of EEG hypersynchronization? As in all scalp EEG recordings, the AS EEG is a superposition of signals from many different cortical processes and regions. To support a state of awareness, there must be a sufficient degree of complex activity in AS during wakefulness (Tononi and Edelman 1998; Tononi *et al.* 2016), with complexity decreasing as consciousness vanishes (i.e. in sleep). Additionally, there must also be a high-voltage delta signal which drowns out the low-voltage activity in the AS EEG, just as one may no longer hear the chatter of conversation over the chanting of the crowd in an arena when both signals temporally coincide (Baars *et al.* 2013). To continue this analogy, the total power of all chatter in the arena, and in the brain, may even exceed the total power of the chanting, which, given its greater coordination, is nonetheless easier to detect (Nunez and Srinivasan 2006) (Fig. 7).

The “chanting” signal in AS, however, must be different from the high-voltage, low-frequency activity typically observed in states of reduced consciousness (Steriade 2000; Alkire *et al.* 2008; Brown *et al.* 2010). This is because the trough of such slow oscillations is believed to be associated with decreased consciousness, in sleep and anesthesia, as the system globally enters a down-state characterized by neuronal hyperpolarization (Buzsáki 2006; Destexhe *et al.* 2007; Wilson 2008; Lewis *et al.* 2012). Yet, in awake children with AS, consciousness does not appear to be periodically interrupted (from an observer’s perspective) as delta oscillations reach their trough. Because a widespread cortical down-state would silence pyramidal cells, it is reasonable to assume that such a global down-state is incompatible with wakeful, volitional behavior and therefore not the mechanism underlying the delta oscillations observed in awake AS children. Instead, as outlined in Fig. 7, down-states in small, local patches of hypersynchronized tissue may project globally to the scalp by volume conduction in AS, resolving the paradox of diffuse delta activity in wakefulness. Indeed, local OFF-periods (extracellular manifestations of down-states) have been observed during wakefulness in sleep deprived rats (Vyazovskiy *et al.* 2011), demonstrating that localized patches of cortical silence during wakefulness are possible. Moreover, in patients with epilepsy, intracranial EEG has also revealed the temporal coincidence of local ON- and OFF-periods in different anatomical regions during NREM sleep (Nir *et al.* 2011;  Sarasso *et al.* 2014; Castelnovo *et al.* 2018). This coexistence of cortical silence and cortical activity is further suggested by the presence of delta oscillations in scalp EEG recorded from healthy adults in REM sleep (Bernardi *et al.* 2019), during which individuals are likely to be conscious and the cortex overall active.

The foregoing lines of evidence suggest that delta oscillations in awake children with AS may reflect restricted occurrences of down-states amid a cortex that is otherwise abuzz with complex activity. It is unclear how many such local generators would be required to explain the AS EEG phenotype. Volume conduction from a single delta generator should result in enormously high delta coherence across the scalp. However, the global delta coherence in AS has recently been shown to be similar to that in TD children during both wakefulness and sleep (den Bakker *et al.* 2018). Thus, if the scenario outlined in Fig. 7 is correct, many such local generators may exist in AS. Local field potential recordings from a mouse model of AS have already shown hypersynchronous delta activity in Layer 4 of V1 while mice are awake, head-fixed, and unanaesthetized (Judson *et al.* 2016; Sidorov *et al.* 2017). Future studies should continue to explore intracranial recordings in mouse models to delineate the spatial extent of cortical hypersynchronization.

Beyond the delta oscillations described above, the awake state AS delta rhythm may also be closely related to delta oscillations involved in inhibiting competing cognitive functions during wakefulness in healthy adults (Dimitriadis *et al.* 2010; Harmony 2013). Perhaps due to their pathologically large amplitude and diffuse nature, delta oscillations in AS might result in a broad and continuous state of cognitive inhibition, as reflected in the profound intellectual disabilities typical of this condition.

## Conclusion, Limitations, and Future Directions

It is important to be mindful of some shortcomings of the present work. First, given the highly abnormal EEG presentation and the short nature of the sleep events in our data, we could not accurately perform sleep staging. Boundaries between sleep and wakefulness were delineated based on visual observation by the EEG technician and, as such, we acknowledge that sleep defined based solely on behavioral criteria is a limitation of this study. Longer sessions (e.g. 24-h recordings) might be better suited to allow an accurate sleep analysis and comparison of different stages. Furthermore, while data known to contain seizures were discarded, not all EEGs were reviewed by a neurologist and the possible inclusion of absence seizures cannot be ruled out entirely. Also, along these lines, arousal was not monitored during wakefulness, though sections known to contain drowsiness were excluded. Next, we were unable to compare sections of sleep EEG that were most and least likely to coincide with dream experiences (i.e. sleep EEG with the lowest and highest ratio of delta to high-frequency power) due to the circular nature of comparing EEG sections that are already defined such that they differ in power. Lastly, while we reported a relative effect of level of consciousness on complexity metrics, a reference cohort of TD children is needed to assess the overall level of complexity present in the AS EEG.

In conclusion, this work resolves the apparent paradox of wakeful, purposefully behaving, children with AS exhibiting an EEG phenotype most typically associated with states of low/no consciousness (Laan and Vein 2005; Vendrame *et al.* 2012; Frohlich *et al.* 2019a). By finding complex brain dynamics that are sensitive to relative level of consciousness even under conditions of extreme cortical hypersychronization, these results suggest that high-voltage, slow EEG activity is not a reliable indicator of unconsciousness. These findings, along with other rare conditions with paradoxical EEG signatures during consciousness (Matsuura *et al.* 1994; Gökyiǧit and Çalişkan 1995; Laan and Vein 2002), warn against reverse inferring low/no consciousness in patients based on delta power (Fingelkurts *et al.* 2013; Lechinger *et al.* 2013). When brain dynamics are severely altered by genetic disorders, epilepsy, or brain injury, complexity-based methods, e.g., perturbational complexity index (Casali *et al.* 2013; Casarotto *et al.* 2016; Comolatti *et al.* 2019), may be better suited for inferring consciousness.

## Data Availability

Data are available from the corresponding author upon reasonable request.

## Supplementary Material

niaa005_Supplementary_Data
