# High-voltage, diffuse delta rhythms coincide with wakeful consciousness and complexity in Angelman syndrome

**DOI:** 10.1093/nc/niaa021

**Published:** 2020-10-05

**Authors:** Joel Frohlich, Lynne M Bird, John Dell’Italia, Micah A Johnson, Joerg F Hipp, Martin M Monti

**Affiliations:** n1 Department of Psychology, University of California Los Angeles, 3423 Franz Hall, Los Angeles, CA, USA; n2 Department of Pediatrics, University of California, San Diego, CA, USA; n3 Division of Genetics/Dysmorphology, Rady Children’s Hospital San Diego, San Diego, CA, USA; n4 Roche Pharma Research and Early Development, Roche Innovation Center Basel, Basel, Switzerland; n5 Department of Neurosurgery, UCLA Brain Injury Research Center, David Geffen School of Medicine, University of California Los Angeles, Los Angeles, CA, USA

*Neuroscience of Consciousness,* Volume 6, Issue 1, 2020, https://doi.org/10.1093/nc/niaa005

In the above article, the authors original stated that spectral power was being normalised by log2(Hz) instead of Hz. The results and conclusions of the paper have not been affected. As such, the sentence *Spectral power was normalized per octave, i.e., log2(Hz), rather than Hz to account for the logarithmic nature of EEG signals (Buzsáki and Draguhn 2004)* on page 3 has now been removed. Additionally, the labels for Fig. 2, Fig. 4, Fig. S1, Fig. S2, Fig. S5, Fig. S7, and Fig. S10 have been changed to *Power log_10_ (μV2/Hz)* and the labels in Fig. S3 and Fig. S8 have been changed to *log_10_ [log2(Hz) μV2/Hz*. These have all been corrected online. The authors apologise for the error.

