# Supplementary material for: High-voltage, diffuse delta rhythms coincide with wakeful consciousness and complexity in Angelman syndrome
Source: Neurosci Conscious. 2020 Jun 14;2020(1):niaa005. doi: 10.1093/nc/niaa005 (PMC7293820; doi:10.1093/nc/niaa005)
Supplement: niaa005_Supplementary_Data [file niaa005_supplementary_data.docx]

**Supplemental material**

**Supplemental methods**

**Signal Complexity**

The original multiscale entropy (MSE) was introduced by Costa and collogues [1] using sample entropy (SampEn) [2], or the tendency for short motifs to reoccur in a signal within a tolerance r (defined as a fixed proportion of the signal’s standard deviation). Here we used mMSE (r = 0.15), an improved version of the original MSE algorithm based on the more robust mSampEn [3], which is less sensitive to r. We implemented mMSE using custom code that patches a commonly cited shortcoming of the original MSE algorithm [4,5] by computing r separately for each timescale. mMSE was computed from 30 s segments with 20 coarse graining scales, thus allowing for as many as 300 samples at the 20^th^ timescale. This number of timescales gives good coverage of the EEG spectrum, with a Nyquist frequency of 100 Hz for the 1st timescale and 5 Hz for the 20^th^ timescale. Following the advice of Grandy and colleagues [6], we rejected all segments that did not include at least 100 valid samples for each timescale.

As an additional complexity measure, we also examined the number of substrings contained in the binarized EEG signal (Lempel and Ziv, 1976) using gMLZ. A multiscale approach to Lempel-Ziv complexity was first advocated by Ibáñez-Molina and colleagues [8], who showed that a dynamic threshold applied with different smoothing windows for different timescales shows better sensitivity to all EEG frequencies than a static threshold obtained from the median of the entire signal, which is biased towards lower EEG frequencies. More recently, however, Yeh and Shi [9] demonstrated that the thresholding approach advocated by Ibáñez-Molina and colleagues may in fact be too biased toward higher EEG frequencies, thus risking overestimates of complexity. As a solution, Yeh and Shi have proposed gMLZ, which uses the same dynamic threshold as the approach given by Ibáñez-Molina and colleagues while also applying a moving median filter with a smaller smoothing window to the signal before binarizing the signal according to its threshold given by a moving median filter with a larger smoothing window. We implemented gMLZ by modifying existing code provided by Hudetz and colleagues that implements the Lempel-Ziv algorithm [10]. Results from a study by Gómez and colleagues show that Lempel-Ziv complexity stabilizes once EEG segments reach a length of ~2000 samples [11], which Ibáñez-Molina and colleagues use as an upper limit for segment size [8]; for this reason, we computed gMLZ from 12 s (2400 sample) EEG segments, thus exceeding the recommendation of Ibáñez-Molina and colleagues in order to afford elbow room for EEG segments with excised artifacts. The gMLZ derived from each EEG segment was normalized according to the number of valid samples n using the quantity n/log_2_(n) [7]. For each timescale, two moving median filters were employed, one to smooth the EEG signal itself and another to compute the dynamic threshold that is used to binarize the signal [9]. We utilized 20 timescales with logarithmically spaced center frequencies 1 – 30 Hz.

**Comparison of Sleep Versus Wakefulness**

Our comparison of data from sleep and wakefulness is informed by the finding that most awakening from NREM sleep are accompanied by reports of dreams [12] and are thus “contaminated” by consciousness. Siclari and colleagues recently found that dreams are most likely to be reported from sleep in which delta power is low and high-frequency (20 – 50 Hz) power is high over a posterior hot zone (PHZ) [13]. Conversely, the same study found that sections of sleep characterized by high delta power and low high-frequency power in the PHZ are likely to coincide with no reportable conscious experience. The ability to stratify sleep according to sections that are more likely and less likely to correspond to conscious experience motivates a more targeted comparison of asleep and awake EEG in AS. In addition to the variance in level of consciousness encountered in sleep, there is large variance in delta amplitude encountered during wakefulness in AS: the awake state AS delta EEG phenotype shows greater dynamic variability (i.e., intermittent bursts of delta activity) across all scalp regions as compared with TD control children [14]. For these reasons, we performed two comparisons of EEG data: 1) a full comparison using all good data from both the awake and the asleep state and 2) a targeted comparison using sections of sleep EEG that are unlikely to coincide with conscious experience (as judged by parietal EEG activity) paired with sections of awake EEG that are especially abnormal as judged by their delta power.

Data sections for the targeted comparison were identified separately for awake and asleep state data by partitioning the time series (awake: delta power; asleep: parietal delta/fast power) into sections that minimize the sum of square difference between each sample and its local mean (MATLAB function: findchangepts). In each case, the maximum number of breaks between data sections was not allowed to exceed the length of usable EEG data in minutes for the given condition. For sleep data, we optimized the ratio of delta power (integrated 1-4 Hz) over high-frequency power (integrated 20 – 45 Hz) averaged across parietal channels (Pz, P3, and P4) from the time-frequency representation of spectral power. For awake data, we optimized delta power (integrated 1-4 Hz) averaged across all channels from the time-frequency representation of spectral power. In each condition, we applied a moving median filter with a 30 s smoothing window and started with the EEG section with the highest mean and continued selecting additional sections with the next highest means until the combined length of all sections met or exceeded a fixed amount determined as a function of the total data length. These fixed amounts were as follows: 30 s (for total data length ≤ 60 s), 60 s (total length ≤ 120 s), 90 s (≤ 300 s), 120 s (total length ≤ 600 s), 180 s (total length ≤ 1200 s), 240 s (total length > 1200 s). EEG data from these sections were then entered into the targeted comparison.

**Supplemental References**

1. Costa M, Goldberger AL, Peng C-K. Multiscale entropy analysis of complex physiologic time series. Phys Rev Lett. 2002;89:068102.

2. Richman JS, Moorman JR. Physiological time-series analysis using approximate entropy and sample entropy. Am J Physiol-Heart Circ Physiol. 2000;278:H2039–49.

3. Xie H-B, He W-X, Liu H. Measuring time series regularity using nonlinear similarity-based sample entropy. Phys Lett A. 2008;372:7140–6.

4. Humeau-Heurtier A. The multiscale entropy algorithm and its variants: A review. Entropy. 2015;17:3110–23.

5. Nikulin VV, Brismar T. Comment on “Multiscale entropy analysis of complex physiologic time series.” Phys Rev Lett. 2004;92:089803.

6. Grandy TH, Garrett DD, Schmiedek F, Werkle-Bergner M. On the estimation of brain signal entropy from sparse neuroimaging data. Sci Rep. 2016;6:23073.

7. Lempel A, Ziv J. On the complexity of finite sequences. IEEE Trans Inf Theory. 1976;22:75–81.

8. Ibáñez-Molina AJ, Iglesias-Parro S, Soriano MF, Aznarte JI. Multiscale Lempel–Ziv complexity for EEG measures. Clin Neurophysiol. 2015;126:541–8.

9. Yeh C-H, Shi W. Generalized multiscale Lempel–Ziv complexity of cyclic alternating pattern during sleep. Nonlinear Dyn. 2018;93:1899–910.

10. Hudetz AG, Liu X, Pillay S, Boly M, Tononi G. Propofol anesthesia reduces Lempel-Ziv complexity of spontaneous brain activity in rats. Neurosci Lett. 2016;628:132–5.

11. Purdon PL, Pierce ET, Mukamel EA, Prerau MJ, Walsh JL, Wong KFK, et al. Electroencephalogram signatures of loss and recovery of consciousness from propofol. Proc Natl Acad Sci. 2013;110:E1142–51.

12. Stickgold R, Malia A, Fosse R, Propper R, Hobson JA. Brain-mind states: I. Longitudinal field study of sleep/wake factors influencing mentation report length. Sleep. 2001;24:171–9.

13. Siclari F, Baird B, Perogamvros L, Bernardi G, LaRocque JJ, Riedner B, et al. The neural correlates of dreaming. Nat Neurosci. 2017;20:872.

14. Sidorov MS, Deck GM, Dolatshahi M, Thibert RL, Bird LM, Chu CJ, et al. Delta rhythmicity is a reliable EEG biomarker in Angelman syndrome: a parallel mouse and human analysis. J Neurodev Disord. 2017;9:17.

**Supplemental Figures**


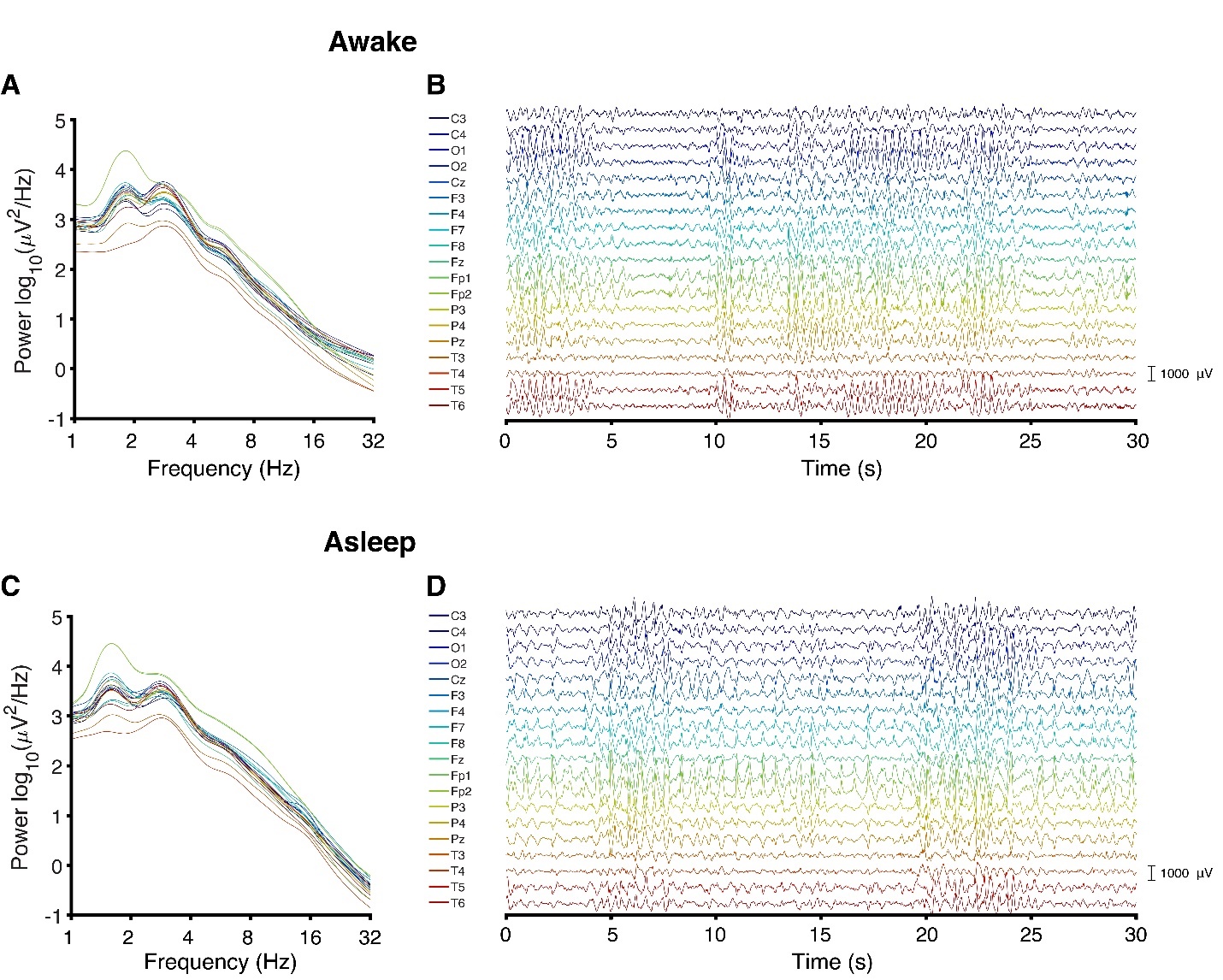


**Figure S1 Additional visualization of EEG from AS participant in Figure 1**. This participant was a 27-month-old girl with AS; she had no seizures and was not taking any medications. The awake state and asleep state EEG strongly resemble each other in both the frequency domain and the time domain. **(A)** Spectral power from awake state EEG. Note the presence of two delta peaks in most channels, one located approximately an octave higher in frequency than the other. **(B)** 30 s of representative EEG from the awake state; this is a longer recording containing the EEG shown in Fig. 1A. Note the presence of high amplitude delta oscillations in virtually all channels and the voltage scale bar at bottom right. **(C)** Spectral power from asleep state EEG. Note the presence of two delta peaks in most channels, one located approximately an octave higher in frequency than the other. **(D)** 30 s of representative EEG from the asleep state; this is a longer recording containing the EEG shown in Fig. 1E. As in the awake state EEG, the asleep state EEG contains high amplitude delta oscillations in virtually all channels; note the voltage scale bar at bottom right.


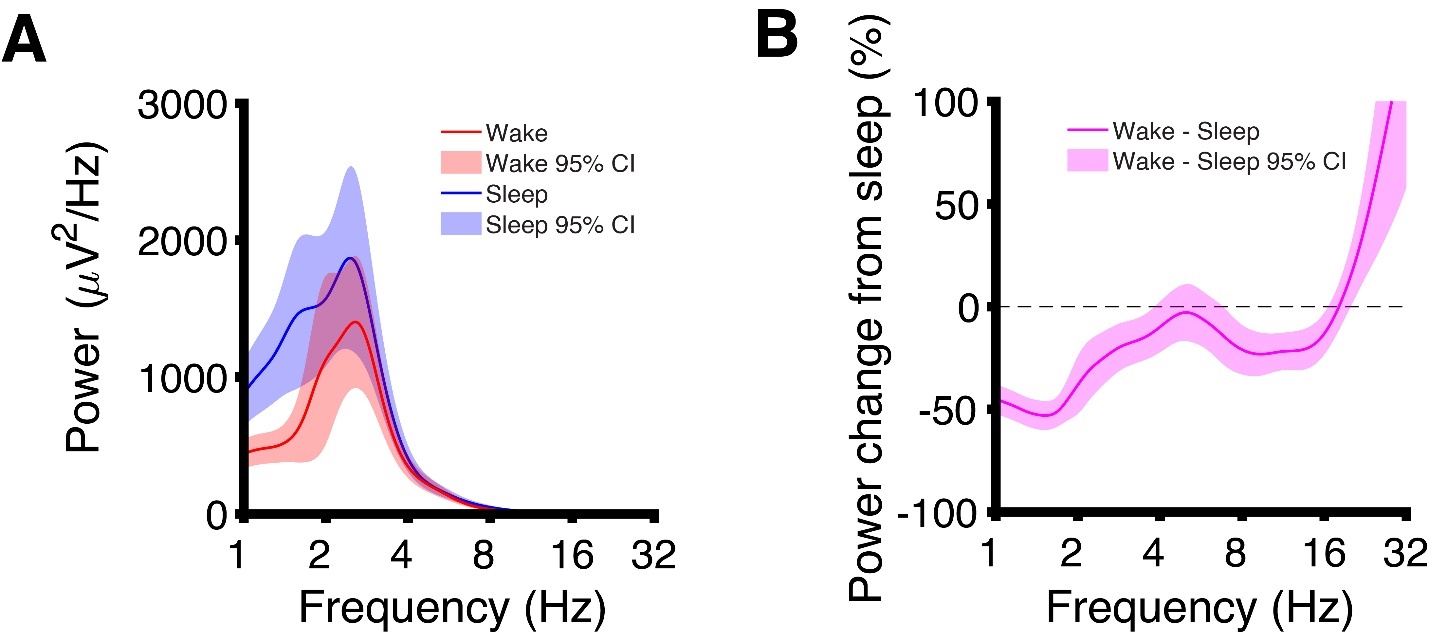


**Figure S2 Alternative visualizations of EEG power and power change from the full comparison. (A)** Channel-averaged (mean ± 95% CI) untransformed EEG power traces demonstrate global maxima in the delta band for both sleep and wakefulness. This untransformed view of the data (without log-scaling) allows for a clearer visualization of the delta band. The delta EEG power in sleep shows a peak which is broader on the left side (with more power in the 1.0 – 1.6 Hz band than in wakefulness as judged by nonoverlapping 95% confidence intervals). Although the delta peak in the asleep state is broad, it features only one local maximum (cf. Fig. S3A). **(B)** Channel-averaged EEG power change (mean ± 95% CI) referenced to sleep. The largest decrease in power is a 53.0% reduction at f = 1.52 Hz.


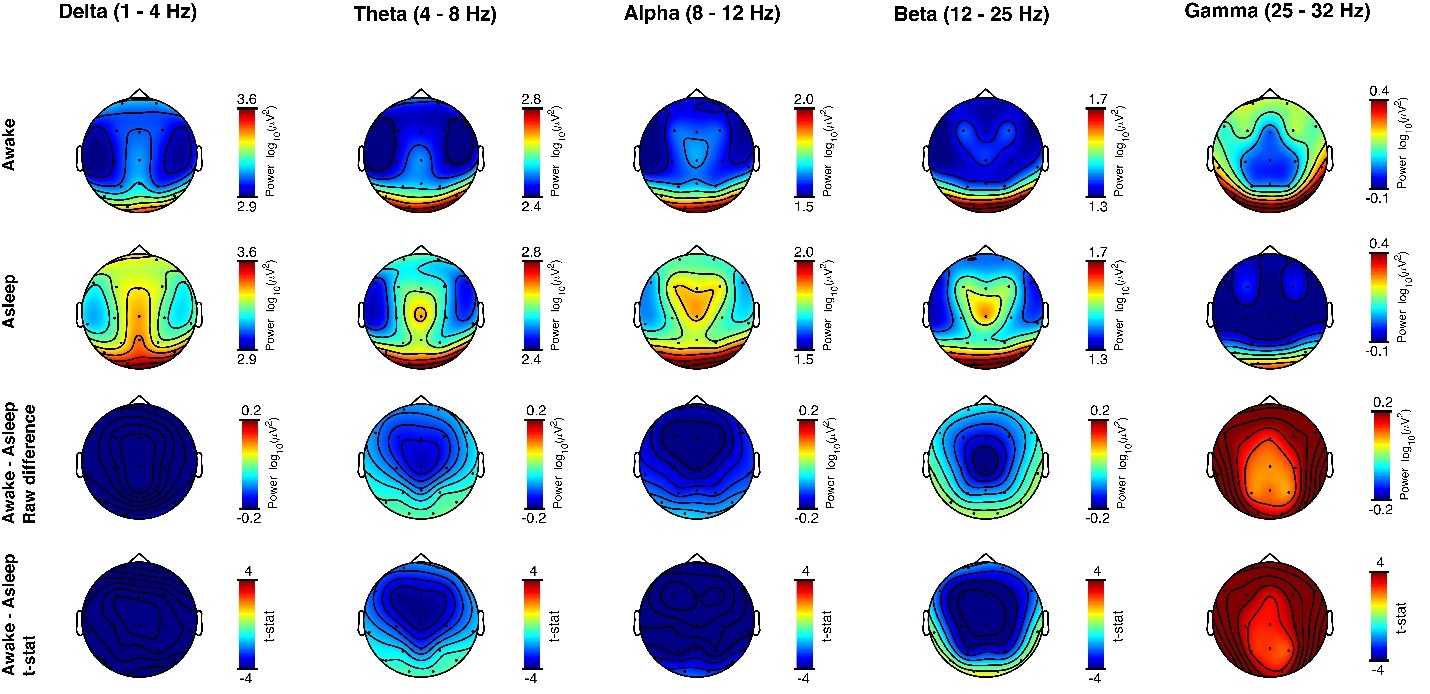


**Figure S3** **Topographic plots of power in the full comparison by frequency band and state.** Scalp plots in the first two rows (first row: awake, second row: asleep) show the integrated power within each frequency band averaged across participants: delta (1 – 4 Hz, first column), theta (4 – 8 Hz, second column), alpha (8 – 12 Hz, third column), beta (12 – 25 Hz, fourth column), and gamma (25 – 32 Hz, fifth column). The third row depicts differences in average power between states (awake – asleep). The fourth row depicts t-statistics from paired samples t-tests of power across states. Note that t-statistics are shown only to visualize differences between EEG frequency bands; permutation cluster statistics were based on t-statistics obtained each at each of 41 frequency bins (wavelets).

**
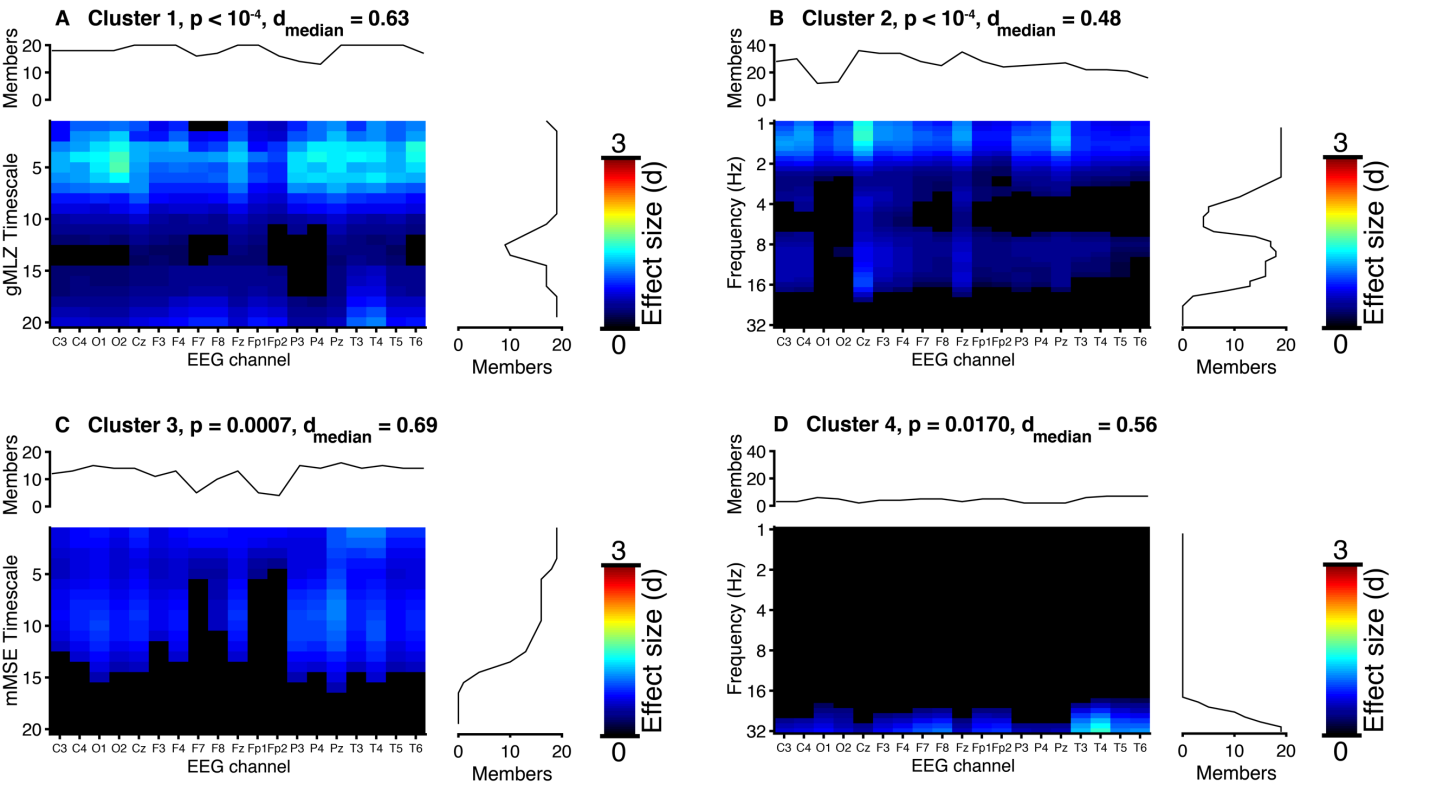
**

**Figure S4 Channel-timescale/frequency space clusters from the full comparison.** Heatmaps reflect the unsigned effect size (Cohen’s d); flanking graphs count cluster membership. Cluster numbers refer to Table 1. Permutation cluster statistics correct for multiple testing across channels and timescales/frequencies, while a Bonferroni correction is used to correct for multiple testing across analyses and EEG measures. Statistical significance is determined using $\alpha$ = 0.0063 (Bonferroni correction). **(A)** Significant gMLZ cluster (greater in wakefulness) covering 90.79% of channel-timescale space (see Fig. 3B). **(B)** Significant power cluster (greater in sleep) covering 62.39% of channel-frequency space (see Fig. 2A). **(C)** Significant mMSE cluster (greater in wakefulness) covering 60.79% of channel-timescale space (see Fig. 3A). **(D)** Power cluster (greater in wakefulness) covering 10.65% of channel-frequency space (not significant after Bonferroni correction).


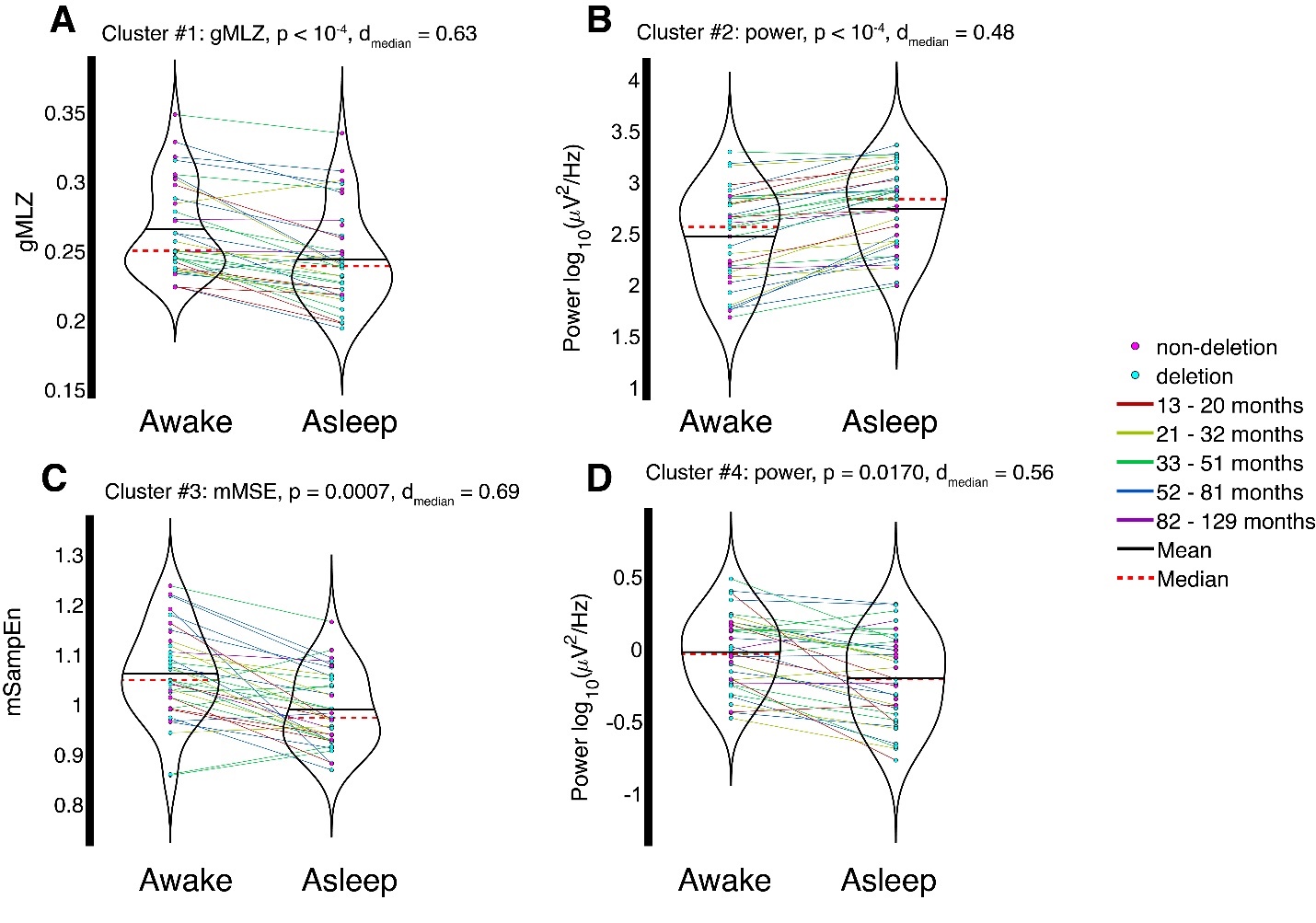


**Figure S5 Violin plots depicting within-cluster averages from the full comparison.** Dots represent the mean taken across all points within channel-frequency or channel-timescale clusters and are colored by the genotype (15q deletion: cyan; non-deletion: magenta) of individual participants. Lines connecting data points between awake (left) and asleep (right) are color coded by participants’ ages (13 – 20 months: red; 21 – 32 months: yellow; 33 – 51 months: green; 52 – 81 months: blue; 82 – 129 months: purple). Because clusters differed greatly in the quantity of the EEG measure, the scaling on the y-axis differs between panels. **(A)** Significant gMLZ cluster (greater in wakefulness, see Fig. 3B). **(B)** Significant power cluster (greater in sleep, see Fig. 2A). **(C)** Significant mMSE cluster (greater in wakefulness, see Fig. 3A). **(D)** Power cluster (greater in wakefulness, not significant after Bonferroni correction).


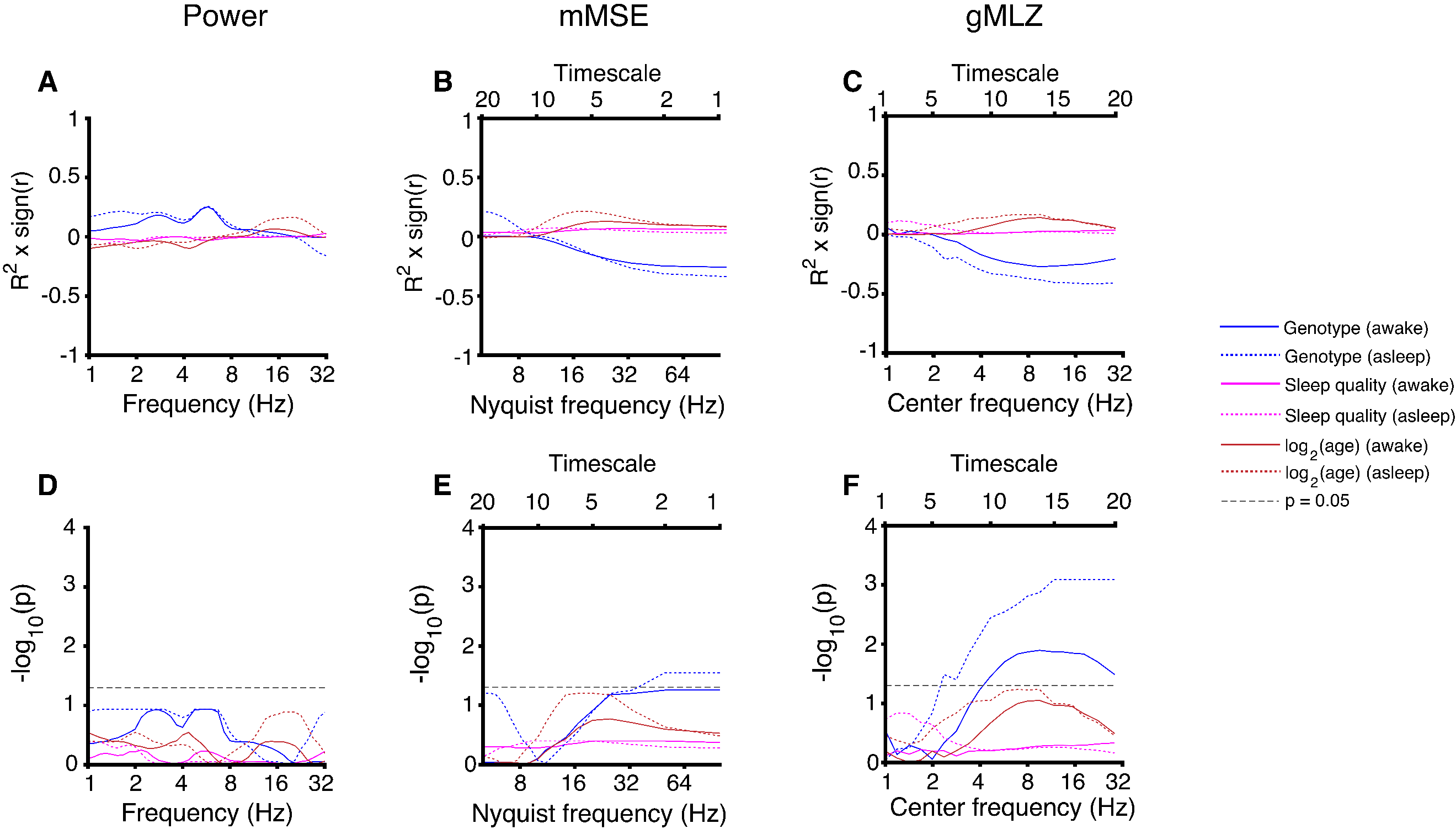


**Figure S6 Correlations between EEG measures and covariates, full comparison.** Correlation coefficients (Pearson’s) were computed to measure the relationship between EEG variables (power, mMSE, and gMLZ) at each frequency/timescale and several covariates (genotype, sleep quality, and age). Correlations were computed for EEG measures in both the awake and asleep state (channel-averaged). For each EEG measure, the statistical significance of correlations was corrected for testing across multiple frequencies/timescales, covariates, and states (awake and asleep) using the false discovery rate (FDR). Genotype (blue) is a categorical variable with two values (deletion and non-deletion). Sleep quality (magenta) is measured as the number of nights per week that the participant sleeps through the night at home as reported by parents. Age (red) is measured as log_2_(months); this transformation is appropriate because larger developmental gains are made at younger ages. Dotted lines indicate relationships with sleep EEG, solid lines indicate relationships with wakeful EEG. Top row: variance in **(A)** spectral power, **(B)** mMSE, and **(C)** gMLZ at each frequency/timescale accounted for by covariates. R^2^ is multiplied by the sign of the correlation coefficient to indicate the directionality of the relationship. Bottom row: statistical significance of relationships between covariates and **(D)** spectral power, **(E)** mMSE, **(F)** and gMLZ. P-values are FDR corrected for multiple testing within each EEG measure. Of the covariates examined, only genotype exceeds the threshold for statistical significance (p = 0.05, corrected, dotted gray line) for two EEG measures: mMSE (sleep only) and gMLZ (wakefulness and sleep). Because our study employed a within-subject design, all covariates are controlled for.


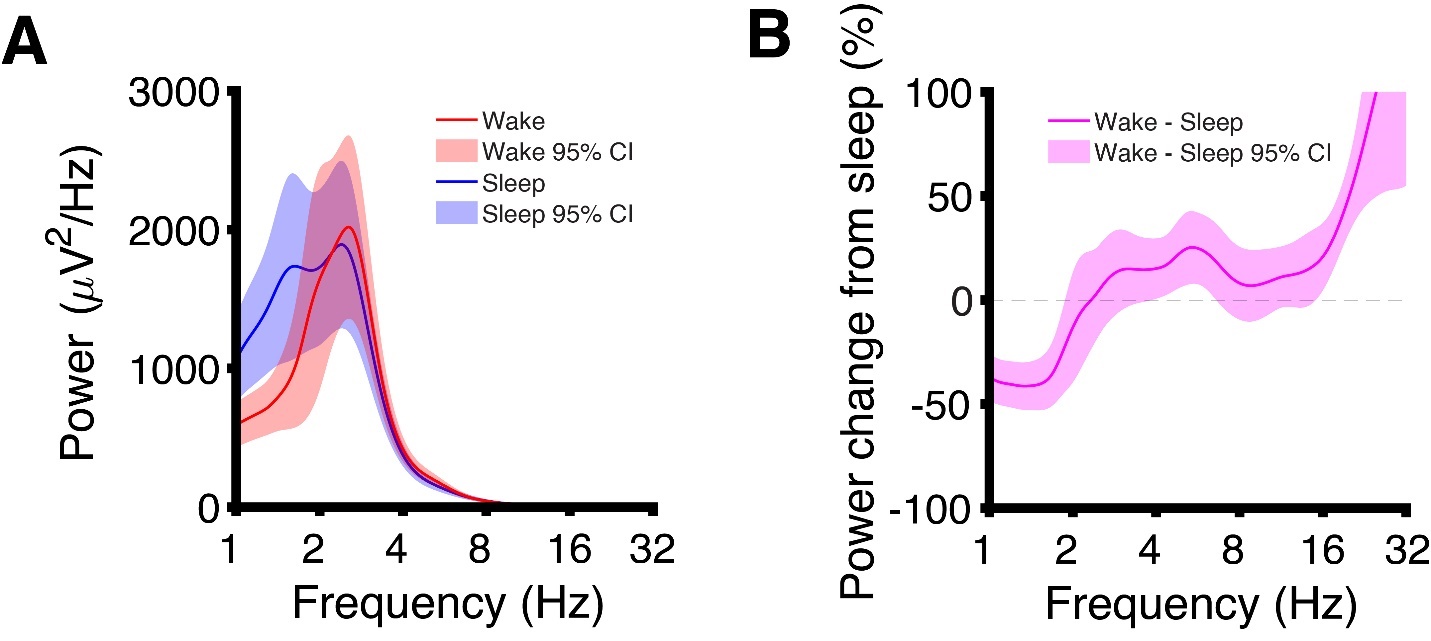


**Figure S7 Alternative visualizations of EEG power and power change from the targeted comparison. (A)** Channel-averaged (mean ± 95% CI) untransformed EEG power traces (targeted comparison) demonstrate global maxima in the delta band for both sleep and wakefulness. This untransformed view of the data (without log-scaling) allows for a clearer visualization of the delta band. The EEG power in sleep shows two peaks in the delta band (f_1_ = 1.62 Hz, f_2_ = 2.40 Hz), whereas only one delta peak is present in wakefulness (f = 2.55 Hz). This suggests two separate oscillatory processes for slow waves in sleep and for the AS EEG phenotype. Note that because the log-transform is a nonlinear transform, peak frequencies differ between untransformed and log-scaled power (cf. Fig. 4A_1_). The asleep EEG exhibits more power in the 1.0 – 1.4 Hz band than the awake EEG as judged by 95% confidence intervals. **(B)** Channel-averaged (mean ± 95% CI) EEG power change referenced to sleep. The largest decrease in power is a 41.3% reduction at f = 1.34 Hz.


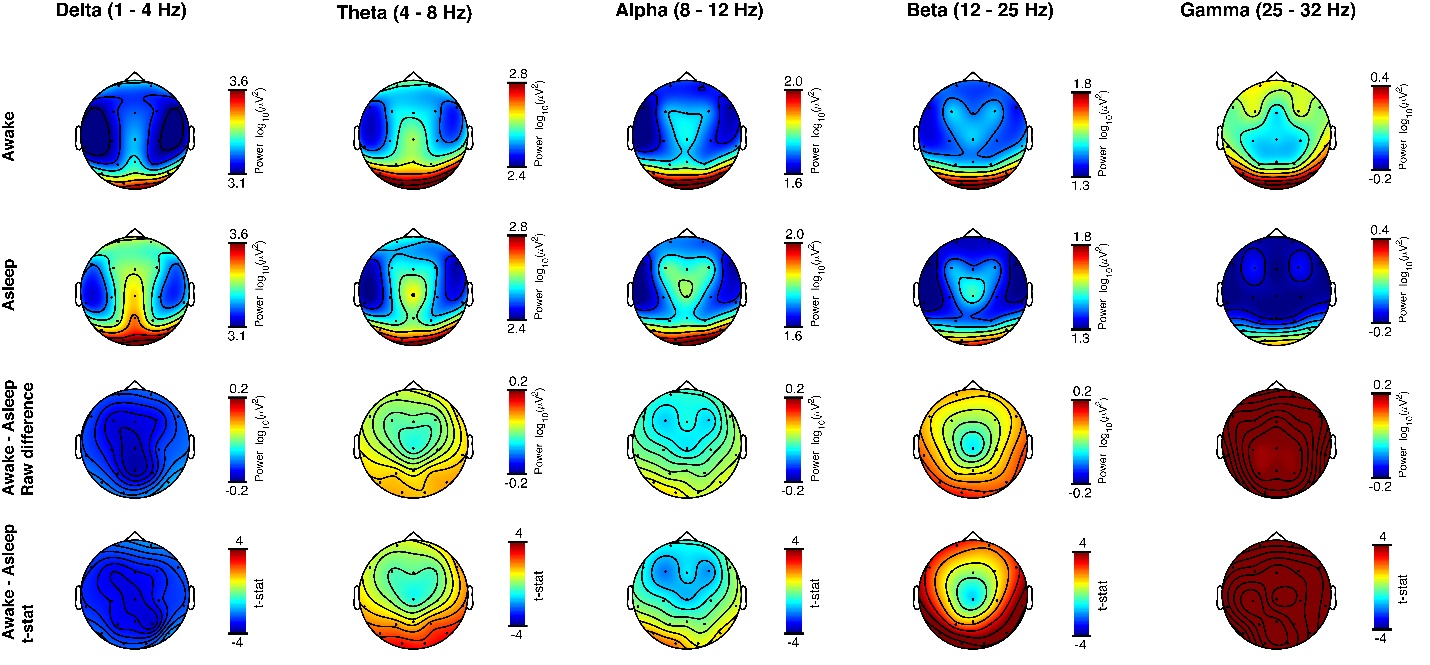


**Figure S8 Topographic plots of power in the targeted comparison by frequency band and state.** Scalp plots in the first two rows (first row: awake, second row: asleep) show the integrated power within each frequency band averaged across participants: delta (1 – 4 Hz, first column), theta (4 – 8 Hz, second column), alpha (8 – 12 Hz, third column), beta (12 – 25 Hz, fourth column), and gamma (25 – 32 Hz, fifth column). The third row depicts differences in average power between states (awake – asleep). The fourth row depicts t-statistics from paired samples t-tests of power across states. Note that t-statistics are shown only to visualize differences between EEG frequency bands; permutation cluster statistics were based on t-statistics obtained each at each of 41 frequency bins (wavelets).

**
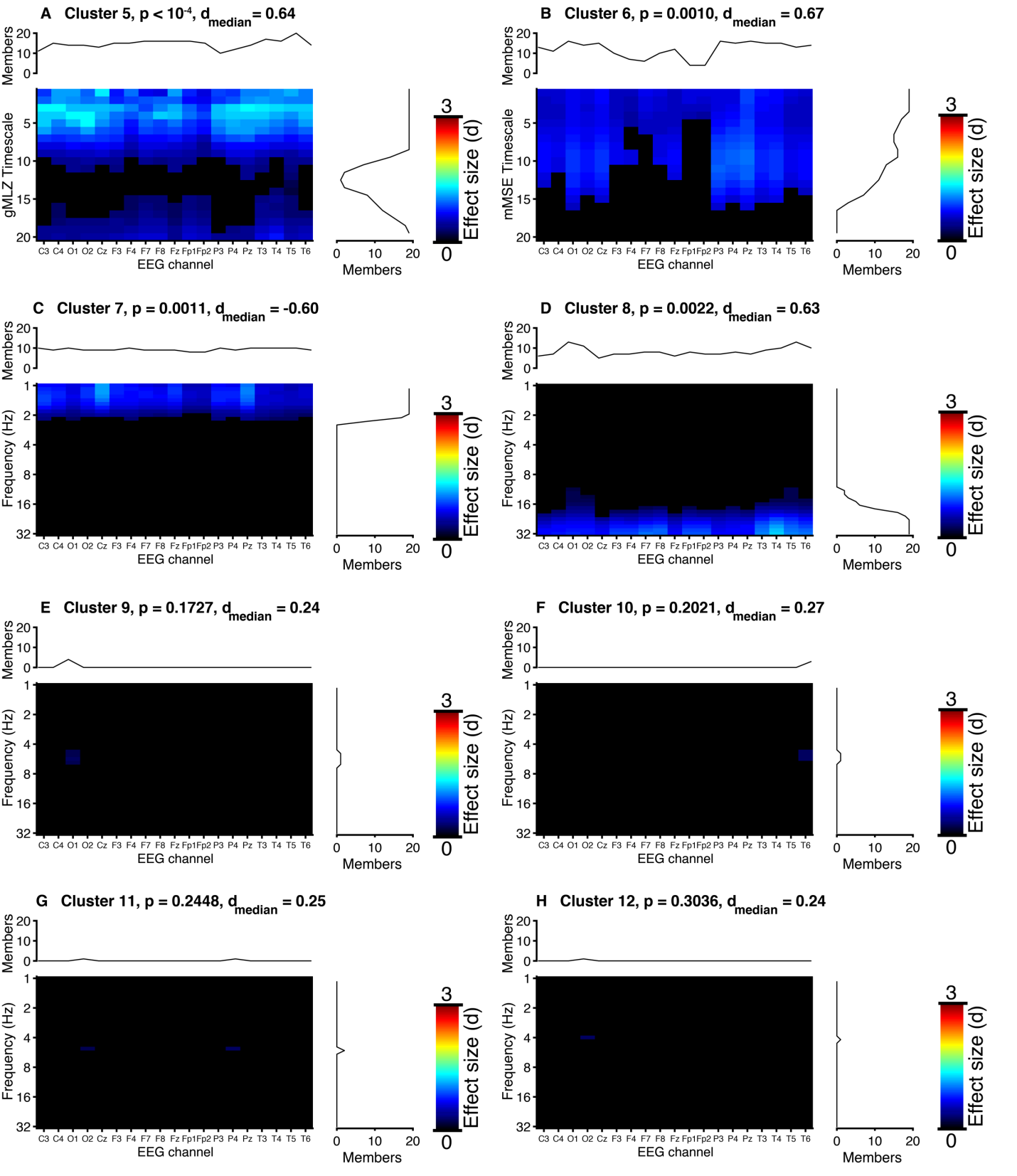
**

**Figure S9 Channel-timescale/frequency-channel space clusters from the targeted comparison.** Heatmaps reflect the unsigned effect size (Cohen’s d); flanking graphs count cluster membership. Cluster numbers refer to Table 1. Permutation cluster statistics correct for multiple testing across channels and timescales/frequencies, while a Bonferroni correction is used to correct for multiple testing across analyses and EEG measures. Statistical significance is determined using $\alpha$ = 0.0063 (Bonferroni correction). **(A)** Significant gMLZ cluster (greater in wakefulness) covering 73.42% of channel-timescale space (see Fig. 5B). **(B)** Significant mMSE cluster (greater in wakefulness) covering 59.47% of channel-timescale space (see Fig. 5A). **(C)** Significant power cluster (greater in sleep) covering 22.72% of channel-frequency space (see Fig. 4A_3,4_). **(D)** Significant power cluster (greater in wakefulness) covering 20.15% of channel-frequency space (see Fig. 4A_5,6_). **(E)** Power cluster (greater in wakefulness, not significant) covering 0.51% of channel-frequency space. **(F)** Power cluster (greater in wakefulness, not significant) covering 0.39% of channel-frequency space. **(G)** Power cluster (greater in wakefulness, not significant) covering 0.26% of channel-frequency space. **(H)** Power cluster consisting of only 1 point (greater in wakefulness, not significant) covering 0.13% of channel-frequency space.


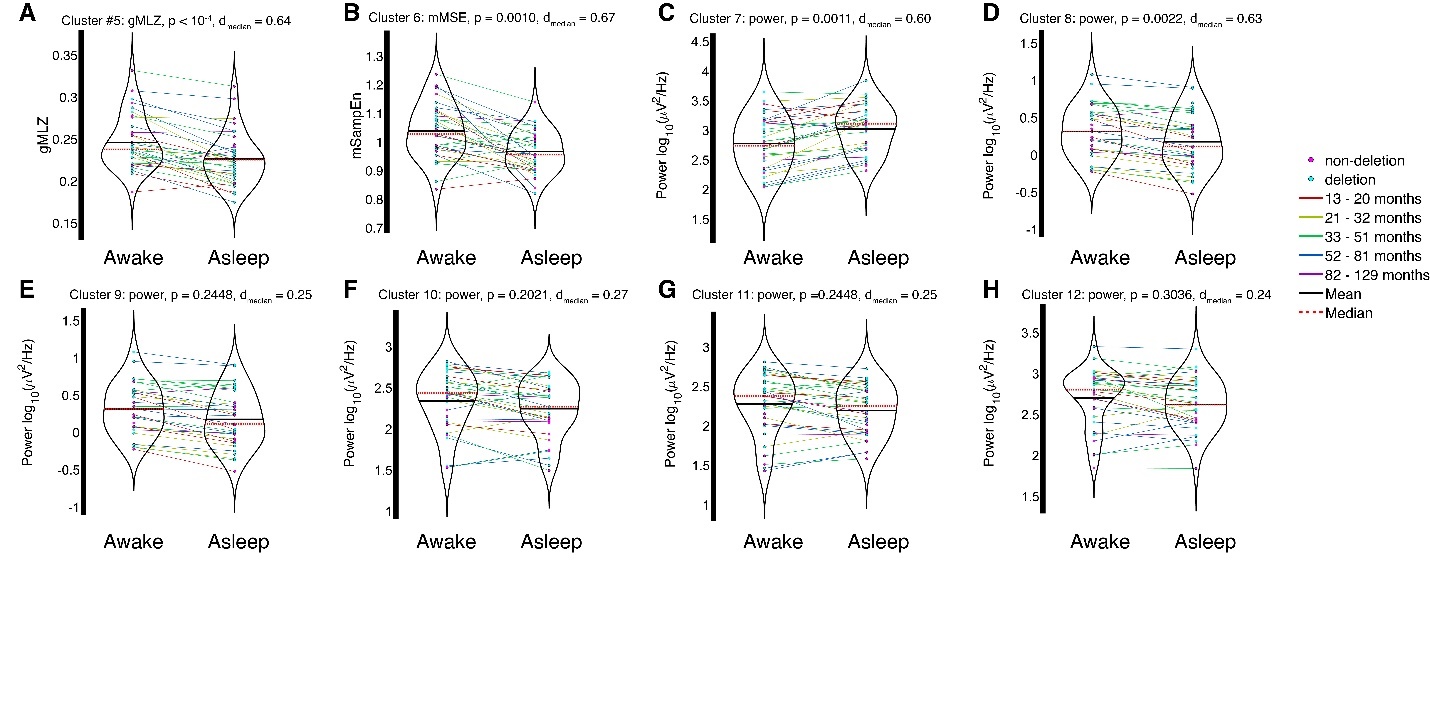


**Figure S10 Violin plots depicting within-cluster averages from the targeted comparison.** Dots represent the mean taken across all points within channel-frequency or channel-timescale clusters and are colored by the genotype (15q deletion: cyan; non-deletion: magenta) of individual participants. Lines connecting data points between awake (left) and asleep (right) are color coded by participants’ ages (13 – 20 months: red; 21 – 32 months: yellow; 33 – 51 months: green; 52 – 81 months: blue; 82 – 129 months: purple). Because clusters differed greatly in the quantity of the EEG measure, the scaling on the y-axis differs between panels. **(A)** Significant gMLZ cluster (greater in wakefulness, see Fig. 5B). **(B)** Significant mMSE cluster (greater in wakefulness, see Fig. 5A). **(C)** Significant power cluster (greater in sleep, see Fig. 4A_3,4_). **(D)** Significant power cluster (greater in wakefulness, see Fig. 4A_5,6_). **(E)** Power cluster (greater in wakefulness, not significant). **(F)** Power cluster (greater in wakefulness, not significant). **(G)** Power cluster (greater in wakefulness, not significant). **(H)** Power cluster consisting of only 1 point in channel-frequency space (greater in wakefulness, not significant).

**
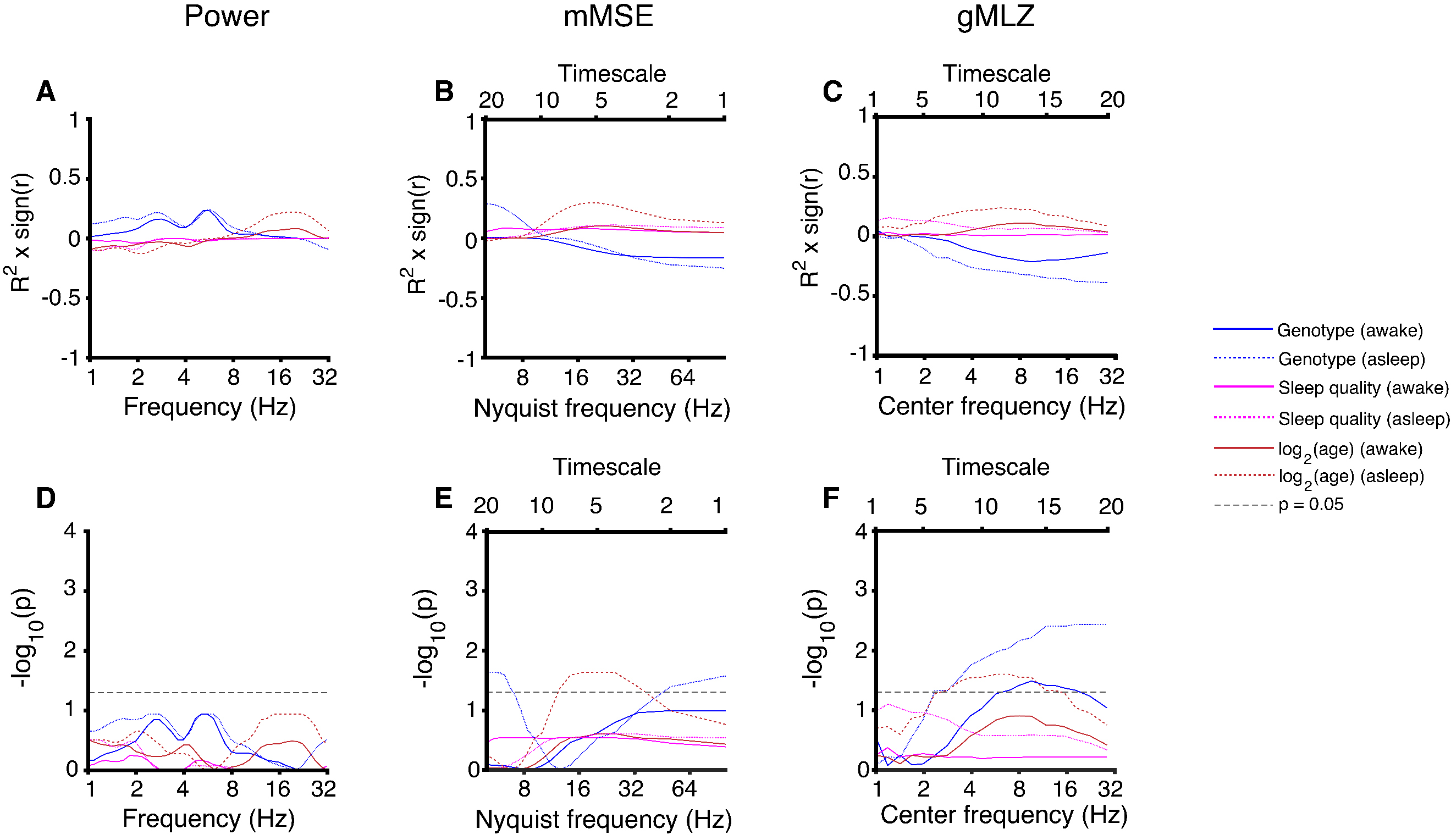
**

**Figure S11 Correlations between EEG measures and covariates, targeted comparison.** Correlation coefficients (Pearson’s) were computed to measure the relationship between EEG variables (power, mMSE, and gMLZ) at each frequency/timescale and several covariates (genotype, sleep quality, and age). Correlations were computed for EEG measures in both the awake and asleep state (channel-averaged). For each EEG measure, the statistical significance of correlations was corrected for testing across multiple frequencies/timescales, covariates, and states (awake and asleep) using the false discovery rate (FDR). Genotype (blue) is a categorical variable with two values (deletion and non-deletion). Sleep quality (magenta) is measured as the number of nights per week that the participant sleeps through the night at home as reported by parents. Age (red) is measured as log_2_(months); this transformation is appropriate because larger developmental gains are made at younger ages. Dotted lines indicate relationships with sleep EEG, solid lines indicate relationships with wakeful EEG. Top row: variance in **(A)** spectral power, **(B)** mMSE, and **(C)** gMLZ at each frequency/timescale accounted for by covariates. R^2^ is multiplied by the sign of the correlation coefficient to indicate the directionality of the relationship. Bottom row: statistical significance of relationships between covariates and **(D)** spectral power, **(E)** mMSE, **(F)** and gMLZ. P-values are FDR corrected for multiple testing within each EEG measure. Of the covariates examined, only genotype and age exceed the threshold for statistical significance (p = 0.05, corrected, dotted gray line) for two EEG measures: mMSE (sleep only) and gMLZ (genotype: wakefulness and sleep; age: sleep only). Because our study employed a within-subject design, all covariates are controlled for.


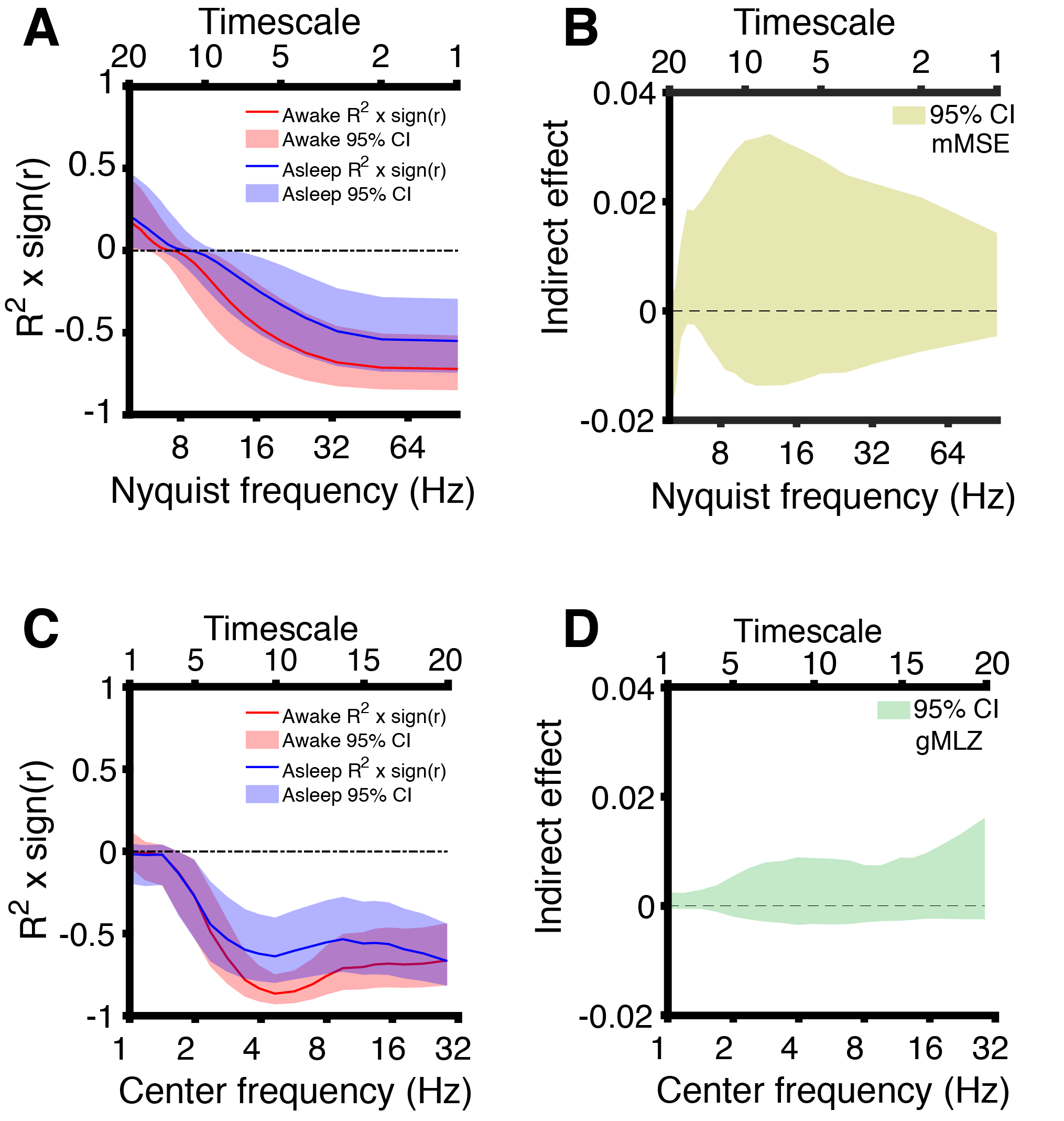


**Figure S12 Effects of sleep on EEG complexity are not mediated by delta power.**

**(A)** Correlations (Pearson’s coefficient, mean ± 95% CI) between mSampEn and delta power computed after averaging across channels.

**(B)** Bootstrapped 95% confidence intervals of the indirect effect of sleep on mMSE mediated by delta power. Delta power did not mediate effects of sleep on mMSE at any timescale, even before correcting for multiple comparisons (p > 0.05 all timescales).

**(C)** Correlations (Pearson’s coefficient, mean ± 95% CI) between gMLZ and delta power computed after averaging across channels.

**(D)** Bootstrapped 95% confidence intervals of the indirect effect of sleep on gMLZ mediated by delta power. Delta power did not mediate effects of sleep on gMLZ at any timescale, even before correcting for multiple comparisons (p > 0.05 all timescales).

**
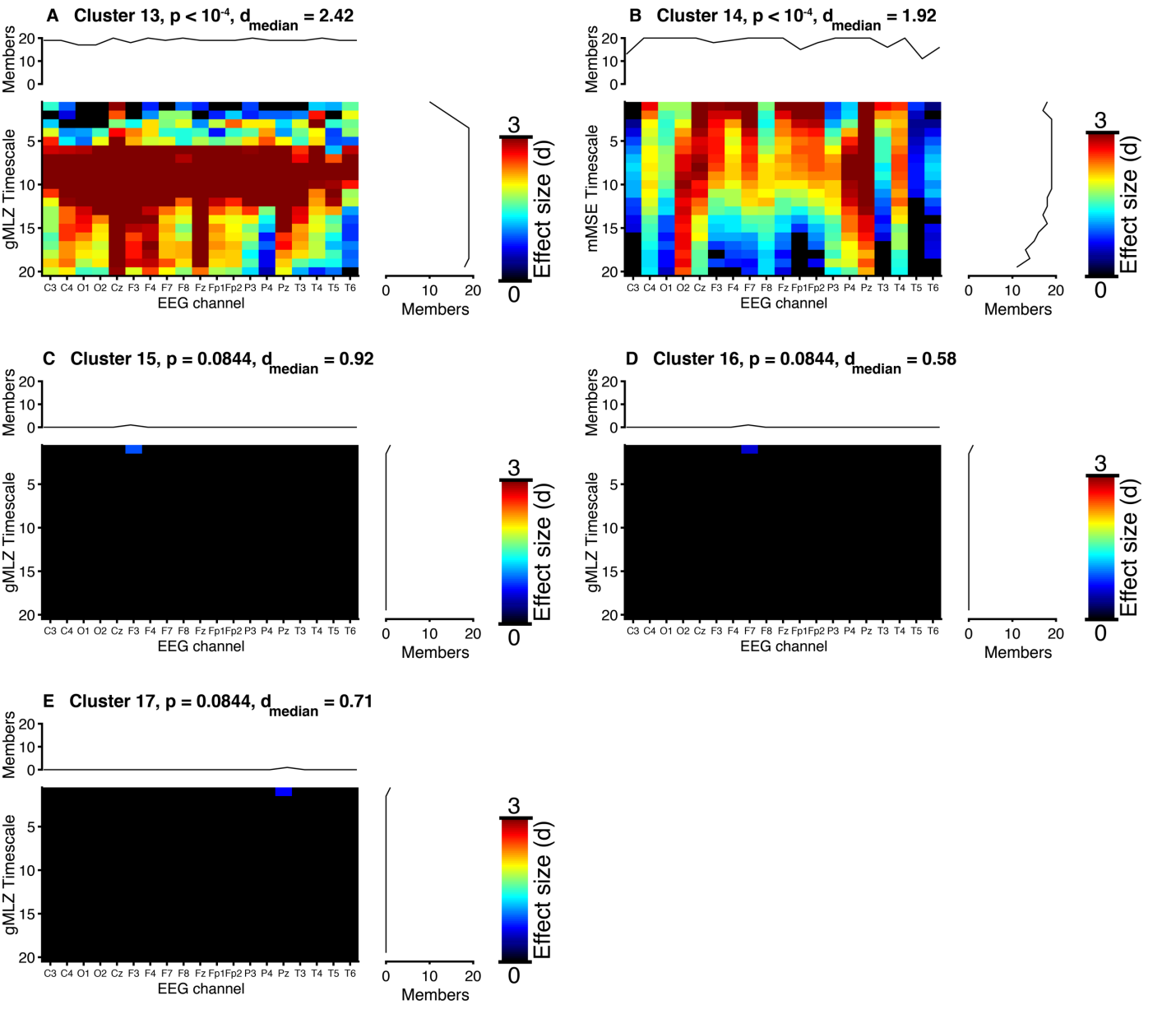
**

**Figure S13 Timescale-channel space clusters from the targeted comparison covarying for delta power.** Heatmaps reflect the unsigned effect size (Cohen’s d); flanking graphs count cluster membership. Cluster numbers refer to Table 1. Permutation cluster statistics correct for multiple testing across channels and timescales/frequencies, while a Bonferroni correction is used to correct for multiple testing across analyses and EEG measures. Statistical significance is determined using $\alpha$ = 0.0063 (Bonferroni correction). **(A)** Significant gMLZ cluster (greater in wakefulness) covering 95.00% of channel-timescale space (see Fig. 6B). **(B)** Significant mMSE cluster (greater in wakefulness) covering 91.05% of channel-timescale space (see Fig. 6A) **(C)** gMLZ cluster consisting of only 1 point (greater in sleep, not significant) covering 0.26% of channel-timescale space. **(D)** gMLZ cluster consisting of only 1 point (greater in sleep, not significant) covering 0.26% of channel-timescale space. **(E)** gMLZ cluster consisting of only 1 point (greater in sleep, not significant) covering 0.26% of channel-timescale space.

**
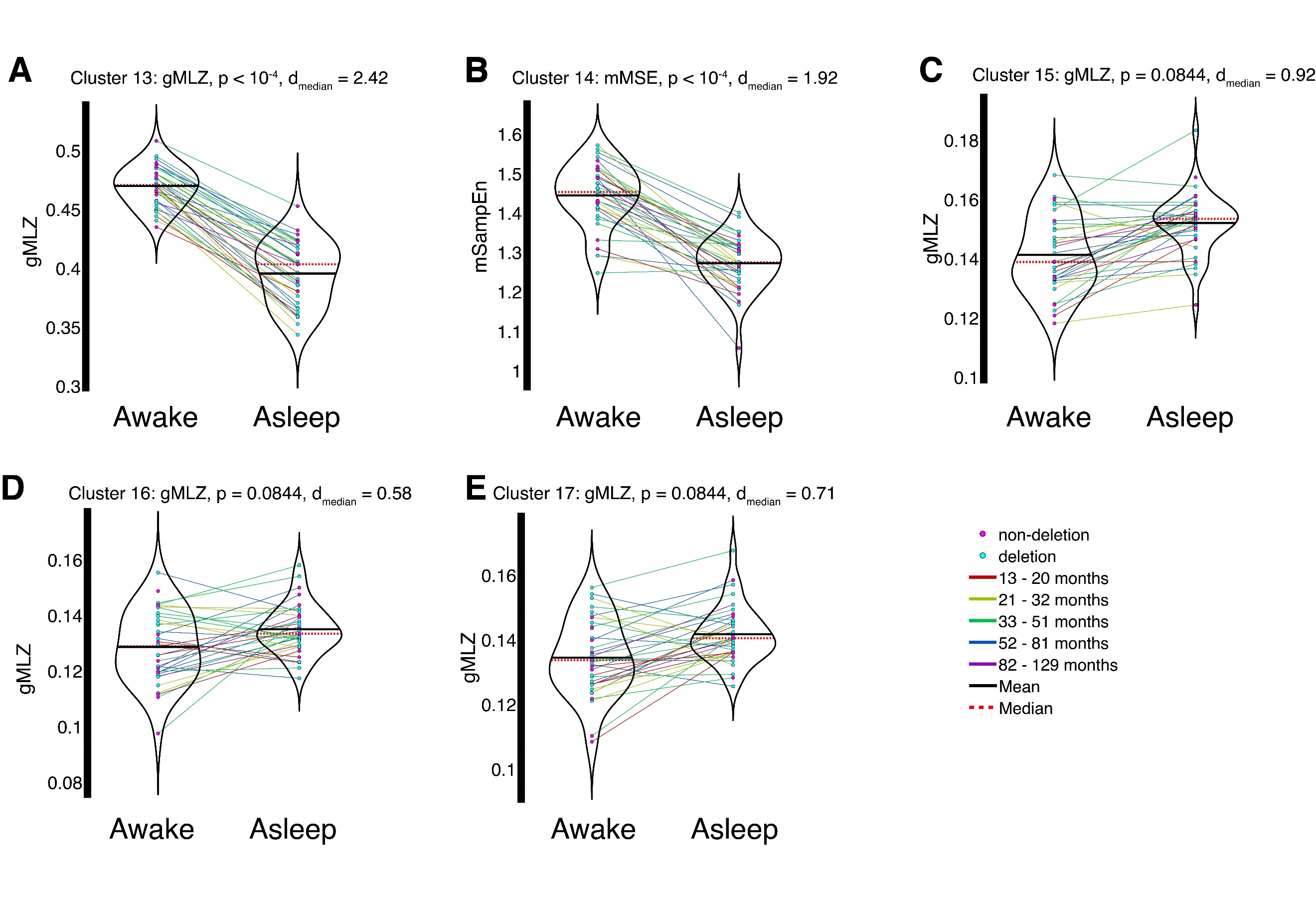
**

**Figure S14 Violin plots depicting within-cluster averages from the targeted comparison covarying for delta power.** Dots represent the mean taken across all points within channel-frequency or channel-timescale clusters and are colored by the genotype (15q deletion: cyan; non-deletion: magenta) of individual participants. Lines connecting data points between awake (left) and asleep (right) are color coded by participants’ ages (13 – 20 months: red; 21 – 32 months: yellow; 33 – 51 months: green; 52 – 81 months: blue; 82 – 129 months: purple). Because clusters differed greatly in the quantity of the EEG measure, the scaling on the y-axis differs between panels. **(A)** Significant gMLZ cluster (greater in wakefulness, see Fig. 6B). **(B)** Significant mMSE cluster (greater in wakefulness, see Fig. 6A) **(C)** gMLZ cluster consisting of only 1 point in channel-timescale space (greater in sleep, not significant). **(D)** gMLZ cluster consisting of only 1 point in channel-timescale space (greater in sleep, not significant). **(E)** gMLZ cluster consisting of only 1 point in channel-timescale space (greater in sleep, not significant).

**Supplemental Tables**

|  | Age (months) | Sex | 15q11.2-q13.1 deletion? | Seizures? | CNS meds? | Awake data, full comparison (s) | Awake data, targeted comparison (s) | Awake sections, targeted comparison | Asleep data, full comparison | Asleep data, targeted comparison (s) | Asleep sections, targeted comparison |
| --- | --- | --- | --- | --- | --- | --- | --- | --- | --- | --- | --- |
| **Participant 1** | 13 | female | Yes | No | No | 1561.5 | 566.6 | 7 | 461.8 | 145.2 | 5 |
| **Participant 2** | 13 | female | No | Yes | Yes | 10019 | 438.1 | 5 | 7362.1 | 253.4 | 5 |
| **Participant 3** | 15 | male | Yes | Yes | No | 372 | 163.5 | 2 | 1635.5 | 266.3 | 9 |
| **Participant 4** | 16 | female | No | Yes | Yes | 664.5 | 193 | 5 | 977.2 | 305.3 | 7 |
| **Participant 5** | 16 | male | Yes | No | No | 470.7 | 149 | 2 | 989.2 | 182.1 | 5 |
| **Participant 6** | 22 | male | Yes | No | Yes | 264.2 | 192.3 | 4 | 1695.1 | 246.1 | 7 |
| **Participant 7** | 24 | male | Yes | Yes | Yes | 615.8 | 221.6 | 3 | 1804.9 | 288.1 | 7 |
| **Participant 8** | 25 | male | Yes | Yes | Yes | 238.5 | 144.5 | 2 | 1585.3 | 261.9 | 9 |
| **Participant 9** | 26 | female | Yes | Yes | Yes | 822.3 | 237.5 | 5 | 319.6 | 148.3 | 2 |
| **Participant 10** | 27 | female | Yes | No | No | 593 | 207 | 2 | 454.7 | 153.6 | 3 |
| **Participant 11** | 29 | male | No | No | Yes | 367.6 | 202 | 2 | 642.2 | 143 | 5 |
| **Participant 12** | 33 | female | Yes | Yes | Yes | 227.1 | 126.8 | 1 | 630.4 | 142.6 | 3 |
| **Participant 13** | 34 | male | Yes | Yes | Yes | 720.8 | 195 | 2 | 1338.4 | 257.5 | 7 |
| **Participant 14** | 36 | female | Yes | No | Yes | 1259 | 350.7 | 4 | 665.3 | 184.6 | 3 |
| **Participant 15** | 37 | male | Yes | Yes | No | 513.1 | 172.8 | 3 | 1774.4 | 241.1 | 7 |
| **Participant 16** | 46 | female | No | No | Yes | 339.6 | 124.4 | 3 | 227.1 | 96.6 | 2 |
| **Participant 17** | 47 | male | No | Yes | Yes | 431.4 | 195.2 | 3 | 1251.1 | 181.1 | 7 |
| **Participant 18** | 47 | male | Yes | Yes | Yes | 1026.1 | 180.9 | 3 | 709.6 | 196.9 | 5 |
| **Participant 19** | 49 | male | No | Yes | Yes | 1048.5 | 208.5 | 5 | 769.9 | 245 | 3 |
| **Participant 20** | 50 | male | Yes | Yes | Yes | 513.2 | 132.7 | 3 | 296.3 | 140 | 3 |
| **Participant 21** | 50 | female | Yes | Yes | Yes | 357 | 146.5 | 3 | 611.9 | 166.6 | 3 |
| **Participant 22** | 51 | male | Yes | Yes | Yes | 1092.7 | 464 | 2 | 693.2 | 126 | 4 |
| **Participant 23** | 52 | male | No | Yes | Yes | 770.1 | 256.2 | 3 | 535.3 | 133.1 | 5 |
| **Participant 24** | 52 | female | No | Yes | Yes | 1670.3 | 245.9 | 4 | 221.4 | 121.4 | 2 |
| **Participant 25** | 52 | female | No | Yes | No | 203.2 | 164.8 | 3 | 173.5 | 88.7 | 2 |
| **Participant 26** | 52 | female | Yes | Yes | Yes | 1972.6 | 344.8 | 3 | 440.6 | 204.8 | 3 |
| **Participant 27** | 54 | female | Yes | Yes | Yes | 732.5 | 296.5 | 5 | 471 | 156.9 | 4 |
| **Participant 28** | 55 | male | Yes | Yes | No | 632.1 | 282 | 3 | 1215.8 | 205 | 6 |
| **Participant 29** | 68 | male | Yes | Yes | Yes | 937.4 | 295 | 2 | 1255.6 | 319 | 8 |
| **Participant 30** | 68 | male | Yes | Yes | Yes | 619.2 | 222.5 | 4 | 1389.1 | 257.3 | 6 |
| **Participant 31** | 78 | female | Yes | Yes | Yes | 450.9 | 203.6 | 5 | 632.4 | 232 | 4 |
| **Participant 32** | 80 | female | Yes | Yes | Yes | 639.5 | 225.2 | 4 | 1349.2 | 249.8 | 5 |
| **Participant 33** | 111 | male | Yes | Yes | Yes | 1167.5 | 284.1 | 4 | 897.7 | 185.2 | 3 |
| **Participant 34** | 118 | male | Yes | Yes | Yes | 1229.3 | 332.1 | 4 | 233.1 | 145.5 | 2 |
| **Participant 35** | 130 | male | No | No | No | 449.9 | 152.8 | 2 | 607.9 | 130.2 | 5 |

**Table S1 AS cohort demographics and data.** Participants are sorted by age and described according to sex, genotype (i.e., the presence of absence of a 15q deletion), seizure status, and medication. The amount of data included in both the full analysis and targeted analysis is also given for each participant. For the targeted analysis, data length is described both in terms of data length (in seconds) and number of continuous data segments. 27 out of 35 (77%) participants were on at least one medication acting on the central nervous system (CNS, includes over-the-counter medications such as melatonin). 27 participants (overlapping with but not identical to the subset on medications) also had a history of seizures at the time of EEG recording.

|  | EEG measure | Comparison | Direction (awake-sleep) | Regressed covariates? | p-value | Cohen’s d  (cluster median) | Cohen’s d  (cluster SD) | Cluster size | percentage | Low freq | High freq | Minimum number of channels | Maximum number of channels |
| --- | --- | --- | --- | --- | --- | --- | --- | --- | --- | --- | --- | --- | --- |
| **Cluster 1a** | gMLZ | Full | Increase | No | < 10^-4^ | 0.96 | 0.27 | 175 | 46.05 | 1 | 6.9 | 1 | 19 |
| **Cluster 1b** | gMLZ | Full | Increase | No | 0.0004 | 0.53 | 0.14 | 77 | 20.26 | 11.8 | 28.6 | 8 | 18 |
| **Cluster 2a** | power | Full | Decrease | No | < 10^-4^ | -0.74 | 0.25 | 228 | 29.27 | 1 | 4 | 1 | 19 |
| **Cluster 2b** | power | Full | Decrease | No | < 10^-4^ | -0.41 | 0.12 | 140 | 17.97 | 5.7 | 19 | 1 | 15 |
| **Cluster 5a** | gMLZ | Targeted | Increase | No | 0.0001 | 0.98 | 0.20 | 138 | 36.32 | 1 | 3.9 | 3 | 19 |
| **Cluster 5b** | gMLZ | Targeted | Increase | No | 0.0013 | 0.50 | 0.08 | 34 | 8.95 | 15.4 | 28.6 | 2 | 13 |

**Table S2 Channel-frequency (power) and channel-timescale (complexity) subclusters isolated using a stricter threshold.** For the fused clusters in Table 1 (Cluster 1, 2, and 5), we isolated each oscillatory or complexity change using a stricter threshold (p = 0.0005) for clustering t-statistics. The resulting new subclusters are named according to their parent cluster in Table 1 (e.g., Cluster 1a and Cluster 1b are both encompassed by Cluster 1 in Table 1). P-values are derived from empirical cluster size distributions using permutation tests. Note that we performed new permutation cluster statistics here with a stricter threshold to learn more about previously identified parent clusters rather than to test new hypotheses. Thus, we did not assess statistical significance of new clusters; however, the original parent clusters were all statistically significant (see Table 1). Effect sizes are reported as Cohen’s d (median and standard deviation across all cluster points).

| Med/Supplement | Occurrences | CNS effects or side effects? | Antiepileptic? |
| --- | --- | --- | --- |
| Total | 204 | 31 | 21 |
| MELATONIN | 23 | TRUE | FALSE |
| OTHER SUPPLEMENTS | 16 | FALSE | FALSE |
| ZANTAC | 14 | FALSE | FALSE |
| KEPPRA | 13 | TRUE | TRUE |
| LAMOTRIGINE | 9 | TRUE | TRUE |
| VALPROIC ACID | 9 | TRUE | TRUE |
| CLONIDINE | 7 | TRUE | FALSE |
| TOPAMAX | 7 | TRUE | TRUE |
| KLONOPIN | 6 | TRUE | TRUE |
| MIRALAX | 5 | FALSE | FALSE |
| PRILOSEC | 5 | FALSE | FALSE |
| ANTIBIOTIC | 4 | FALSE | FALSE |
| BENADRYL | 4 | TRUE | FALSE |
| DIASTAT | 4 | TRUE | TRUE |
| FLOVENT | 4 | FALSE | FALSE |
| LEVODOPA/CARBIDOPA | 4 | TRUE | FALSE |
| PHENOBARBITAL | 4 | TRUE | TRUE |
| REGLAN | 4 | FALSE | FALSE |
| ALBUTEROL | 3 | FALSE | FALSE |
| ATIVAN | 3 | TRUE | TRUE |
| BETAINE | 3 | FALSE | FALSE |
| LAXATIVE/STOOL SOFTENER | 3 | FALSE | FALSE |
| PREVACID | 3 | FALSE | FALSE |
| PULMICORT | 3 | FALSE | FALSE |
| CLOBAZAM | 2 | TRUE | TRUE |
| CLORAZEPATE | 2 | TRUE | TRUE |
| DEPAKENE | 2 | TRUE | TRUE |
| FOLIC ACID | 2 | FALSE | FALSE |
| QVAR | 2 | FALSE | FALSE |
| SINGULAIR | 2 | FALSE | FALSE |
| TAGAMET | 2 | FALSE | FALSE |
| VENTOLIN | 2 | FALSE | FALSE |
| VITAMIN B12 | 2 | FALSE | FALSE |
| ZONEGRAN | 2 | TRUE | TRUE |
| BACLOFEN | 1 | TRUE | TRUE |
| BUDESONIDE | 1 | FALSE | FALSE |
| CARBAMAZEPINE | 1 | TRUE | TRUE |
| CARBATROL | 1 | TRUE | TRUE |
| CARNITINE | 1 | TRUE | FALSE |
| CBD OIL | 1 | TRUE | TRUE |
| CREATINE | 1 | TRUE | FALSE |
| CURCUMIN | 1 | FALSE | FALSE |
| CYPROHEPTADINE | 1 | TRUE | FALSE |
| DIAZEPAM | 1 | TRUE | TRUE |
| EYE DROPS | 1 | FALSE | FALSE |
| GABAPENTIN | 1 | TRUE | TRUE |
| HYDROXYZINE | 1 | FALSE | FALSE |
| METAFOLIN | 1 | FALSE | FALSE |
| NASONEX | 1 | FALSE | FALSE |
| NEXIUM | 1 | FALSE | FALSE |
| ONFI | 1 | TRUE | TRUE |
| PERIACTIN | 1 | TRUE | FALSE |
| RISPERDAL | 1 | TRUE | FALSE |
| SALINE NOSE DROPS | 1 | FALSE | FALSE |
| TRAZODONE | 1 | TRUE | FALSE |
| VALIUM | 1 | TRUE | TRUE |
| VIMPAT | 1 | TRUE | TRUE |
| ZYRTEC | 1 | FALSE | FALSE |

**Table S3 Summary of medications.** Information on current medications and supplements was obtained from families of participants. Because comparisons in our study were all within-participant (awake vs asleep from the same EEG session), all potential medication effects are controlled for. A total of 204 medications and supplements were recorded from study participants, summarized above as 58 unique categories. Of these 58 categories of medications and supplements, 31 had CNS effects or CNS side-effects and 21 were antiepileptic drugs.
